# The Evidence Base for Interventions Delivered to Children in Primary Care: An Overview of Cochrane Systematic Reviews

**DOI:** 10.1371/journal.pone.0023051

**Published:** 2011-08-01

**Authors:** Peter J. Gill, Kay Yee Wang, David Mant, Lisa Hartling, Carl Heneghan, Rafael Perera, Terry Klassen, Anthony Harnden

**Affiliations:** 1 Department of Primary Care Health Sciences, University of Oxford, Oxford, United Kingdom; 2 Faculty of Medicine and Dentistry, University of Alberta, Edmonton, Canada; 3 Department of Pediatrics, Alberta Research Centre for Health Evidence, University of Alberta, Edmonton, Canada; 4 Department of Pediatrics, Cochrane Child Health Field, University of Alberta, Edmonton, Canada; 5 Department of Pediatrics and Child Health, Manitoba Institute of Child Health, University of Manitoba, Winnipeg, Canada; Children's Hospital of Eastern Ontario, Canada

## Abstract

**Background:**

As a first step in developing a framework to evaluate and improve the quality of care of children in primary care there is a need to identify the evidence base underpinning interventions relevant to child health. Our objective was to identify all Cochrane systematic reviews relevant to the management of childhood conditions in primary care and to assess the extent to which Cochrane reviews reflect the burden of childhood illness presenting in primary care.

**Methodology/Principal Findings:**

We used the Cochrane Child Health Field register of child-relevant systematic reviews to complete an overview of Cochrane reviews related to the management of children in primary care. We compared the proportion of systematic reviews with the proportion of consultations in Australia, US, Dutch and UK general practice in children. We identified 396 relevant systematic reviews; 358 included primary studies on children while 251 undertook a meta-analysis. Most reviews (n = 218, 55%) focused on chronic conditions and over half (n = 216, 57%) evaluated drug interventions. Since 2000, the percentage of pediatric primary care relevant reviews only increased by 2% (7% to 9%) compared to 18% (10% to 28%) in all child relevant reviews. Almost a quarter of reviews (n = 78, 23%) were published on asthma treatments which only account for 3–5% of consultations. Conversely, 15–23% of consultations are due to skin conditions yet they represent only 7% (n = 23) of reviews.

**Conclusions/Significance:**

Although Cochrane systematic reviews focus on clinical trials and do not provide a comprehensive picture of the evidence base underpinning the management of children in primary care, the mismatch between the focus of the published research and the focus of clinical activity is striking. Clinical trials are an important component of the evidence base and the lack of trial evidence to demonstrate intervention effectiveness in substantial areas of primary care for children should be addressed.

## Introduction

In both developed and developing countries, the clinical care of children accounts for a large proportion of primary care. For example, in the UK, over 95% of NHS clinical contacts are made in general practice with over 300 million taking place each year,[1] and consultations with children under 15 years represent 18% of all primary care consultations in the UK, 17% in the US and 12% in Australia [2], [3], [4]. A 10,000-patient general practice will expect to have 1,500 children under 16 years registered [2].

There are concerns about the quality of primary care delivered to children in the UK due to episodes highlighted by the confidential enquiry into child deaths and increases in Accident and Emergency presentations, short admissions and unplanned hospitalizations [5], [6]. Similar concerns about the quality of ambulatory care for children have been documented in the US [7]. Globally, more than 10.6 million children under 5 years of age die each year, mostly from preventable and treatable causes, including pneumonia, diarrhea, malaria, injuries, HIV/AIDS, measles and malnutrition [8], [9], [10], [11]. The high level of child mortality in poor countries indicates that high quality evidence based provision of child healthcare is an even greater problem.

However, measuring the quality of care of children is challenging due to the lack of pediatric quality measures [12]. The first step to developing a framework to evaluate and improve the quality of care of children is the identification of the evidence base for interventions relevant to child health and primary care. Systematic reviews are the most comprehensive syntheses of information in healthcare [13] and represent a logical starting point to identify high quality evidence relevant to a broad clinical topic.

The number of systematic reviews published annually has steadily increased over the past decade [14]. In 2007 alone, 2,500 systematic reviews of research were published, with roughly 20% of them produced through the Cochrane Database of Systematic Reviews (CDSR) [15]. The primary aim of the Cochrane Collaboration is to make available up-to-date syntheses of reliable evidence regarding the benefits and risks of healthcare interventions [16]. Cochrane systematic reviews are widely regarded as methodologically rigorous and of higher quality than non-Cochrane systematic reviews [16], [17], [18], [19], [20]. The CDSR therefore represents a sample of high quality studies to better understand the current state of the evidence base.

A previous review described the evidence available from child-relevant systematic reviews in the CDSR, yet it did not determine the evidence base relevant to primary care [16]. In addition no previous studies have compared the scope and number of systematic reviews published with the burden of illness in general practice. Therefore, we completed an overview of systematic reviews in the CDSR relevant to the management of childhood conditions in primary care and compared the topics and number of systematic reviews with the burden of childhood illness in primary care. These efforts provide a framework for researchers and policy makers to identify avoidable waste in the production of research evidence [15] and will lead to further initiatives in the CDSR and beyond to improve the quality of evidence for children in primary care.

## Methods

### Ethics

Data for this study was acquired through previously published work, no patient or hospital data was accessed. Therefore, written consent and institutional ethical review was not required for this research. The PRISMA checklist and flow diagram are available as supporting information; see PRISMA Checklist S1 and PRISMA Flow Diagram S1.

### Searching

We identified eligible systematic reviews from the Cochrane Child Health Field Reviews Register, a register of all child health relevant systematic reviews published in the CDSR. The search terms used to develop the register are outlined in Appendix S1. The register includes all Cochrane systematic reviews that intended to include children (0–18 years of age) or studied an intervention intended to improve the health and wellbeing of children, consistent with those of the Cochrane Child Health Field Trials Register [16], including reviews that included both children and adult participants. The original register included all studies identified in Issue 2, 2009 of the CDSR and was updated in Issue 8, 2010. We did not include Cochrane protocols.

### Study selection

Three reviewers (PG, KW and AH) independently screened a sample of 100 abstracts to pilot and refine the inclusion criteria. Subsequently, two reviewers (PG and KW) independently screened all potentially relevant studies for inclusion. Disagreement was resolved by consensus or consultation with a third reviewer (AH or DM) at all stages. We included systematic reviews regardless of the study designs included. We excluded studies that focused only on children aged less than one month as we felt that the majority of neonatal interventions would be delivered in secondary and tertiary care.

Our definition of ‘primary care setting’ included general or family practice, ambulatory care, pediatric outpatient clinics, pediatric assessment units or emergency departments. We included systematic reviews that were relevant to the management of childhood conditions in primary care or which evaluated interventions that could be administered in the primary care setting by a healthcare provider (screening for early detection of disease, diagnosis of conditions, initiation of treatment, referral for secondary care and ongoing monitoring in primary care). To focus on topics specific to general practice, we excluded school based interventions, specialist nurses, interventions delivered during pregnancy, public health programs and orthodontic and specialist dental procedures.

### Data abstraction

The Cochrane Child Health Field provided data from systematic review register in a Microsoft Excel sheet. The data available in the register for each systematic review are described elsewhere [16] and include general review characteristics, characteristics of included studies and methodological approaches. We updated the register and classified included systematic reviews according to type of care (i.e. acute, chronic or preventative) [7] and applicability to developing or developed countries (defined by the United Nations, which includes Europe, Canada, the USA, Australia, New Zealand, and Japan). One reviewer (PG) extracted data which were checked by a second (KW). Disagreement was resolved by consensus or consultation with a third reviewer (AH or DM) at all stages.

### Burden of illness

We identified burden of illness data from publically available datasets for Australia, the Netherlands, the UK and the US which were classified according to the International Classification of Disease revision 9 (ICD-9) or the International Classification of Primary Care 2^nd^ Edition (ICPC-2). In Australia, the Bettering the Evaluation and Care of Health (BEACH) Project provides an annual database of approximately 100,000 general practice records classified by ICPC-2 [21]. The Royal College of General Practitioners Weekly Returns Service (WRS) is a sentinel general practice network located across England and Wales that routinely monitors disease episodes from every consultation on a weekly basis by ICD-9 [2], [22], [23]. In the US, the Centers for Disease Control and Prevention and the National Center for Health Statistics publish periodic reports on ambulatory healthcare visits from specific time periods [3]. Lastly, we obtained Dutch data on childhood morbidity in general practice through a literature search [24]. The ages included in the dataset ranged from 0–15 to 0–17 years. Where possible, we used estimates over a 3-year period to reduce the possibility of year-to-year fluctuations. The codes reflect primary diagnosis (US), last diagnosis in consultation (the Netherlands) or any diagnosis (Australia and UK) [2], [3], [21], [24], [25]. Estimates of the burden of illness data are approximate rather than precise due to the limitations of the disease coding systems and availability of data. Further details are outlined in Appendix S2.

ICD-9 has many limitations in providing useful clinical data in primary care due to the structure of the axes (e.g. diagnostic entities can with equal logic be classified in more than one chapter) [26],[27]. Several specific diagnostic codes must be combined to generate clinically meaningful data (see Appendix S2). ICPC-2 was developed for use in primary care and classifies clinical activity in the domains of General/Family Practice, taking into account the patient's reason for encounter, the problems/diagnosis managed and interventions [28]. We categorized all systematic reviews by ICPC-2 and by ICD-9 Disease Related Groups. When comparing systematic reviews to burden of illness data, we excluded reviews relevant to developing countries only, as the burden of illness data is specific to developed countries. Due to the structural differences in coding systems, we present the findings separately by ICPC-2 chapters and by ICD-9 (specific diagnostic categories) where relevant. We also compared the number of systematic reviews relevant to low-income countries with the major causes of death in children under 5 years of age other than neonates in Africa and Southeast Asia [10]. Data were analyzed descriptively in PASW Statistics Version 18.0 using frequencies and proportions.

## Results

### Flow of included studies

**Figure 1:**
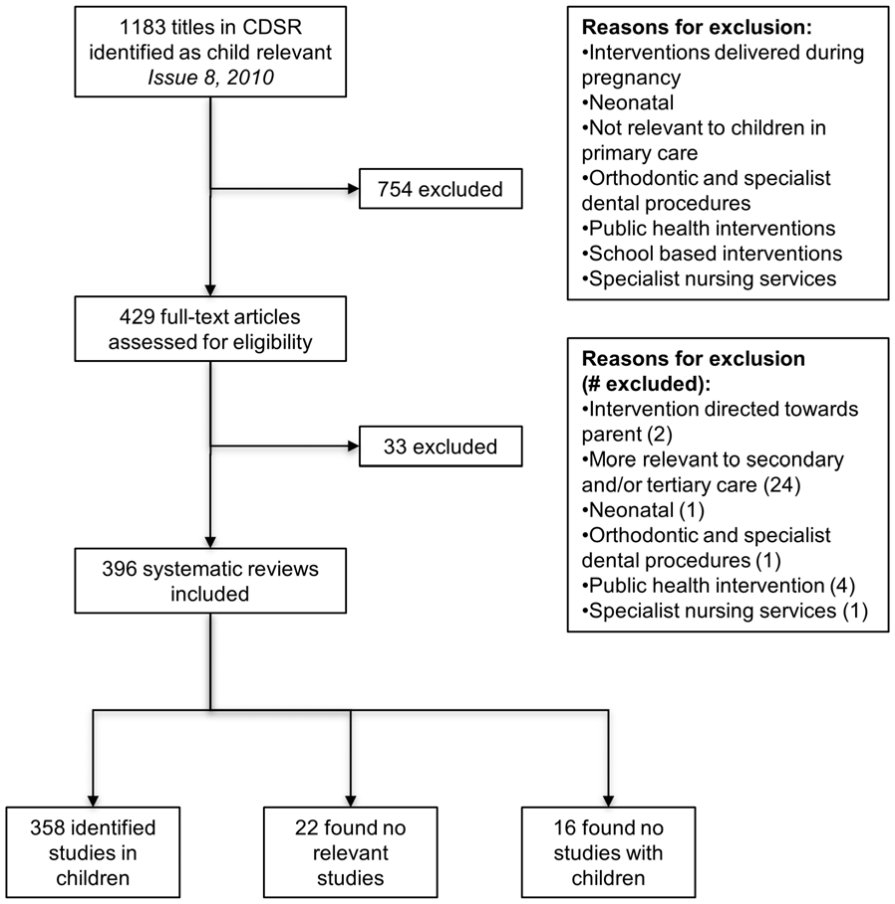
Flow chart of study selection.

From 1,183 systematic reviews we excluded 787 systematic reviews that did not fulfill the inclusion criteria (Figure 1). We included 396 systematic reviews; a full list of included systematic reviews is included in Table S1.

### Study characteristics

Of the 396 systematic reviews, 38 (10%) found no relevant studies (n = 22) or only found studies whose participants did not include children (n = 16) (Table 1). There were 216 systematic reviews (57%) of drug interventions and 14 (4%) which evaluated a drug and a complex or surgical intervention. Fifty-five (14%) systematic reviews focused exclusively on developing countries. Of the 6,022 primary studies included in the reviews, 3,385 included children only and 899 included children and adults.

**Table 1:** Characteristics of included Cochrane systematic reviews.

Characteristic		N (%)*
Total		396 (100)
Authors identified studies/participants for inclusion†		358 (90.4)
Total number of primary studies included^a^	Child only participants, total	3,385
	Child and adult participants, total	899
	Adult only participants, total	1,288
	Age not stated, total	450
Number of studies included per systematic review	All included studies, median (range)	10 (1–144)
	Child only studies, median (range)	5 (0–144)
Number of participants included per systematic review	All included studies, median (range)‡	1,428 (3–4,897,966)
	Child only participants, median (range)§	540 (0–3,141,224)
Methodological considerations	Meta-analysis completed	251 (70.1)
	Children (<18) only in meta-analysis	172 (48.0)
Date since last updated**	Before 2008	222 (56.3)
	Before 2005	66 (16.8)
	Before 2002	22 (5.6)
	Before 1999	2 (0.5)
Single intervention evaluated		343 (95.8)
Specific category of intervention††	Drug	216 (57.4)
	Complex	43 (11.4)
	Natural health product	34 (9.0)
	Device	20 (5.3)
	Vaccine	20 (5.3)
	Clinical	16 (4.3)
	Other	16 (4.3)
	Surgery	11 (2.9)
Type of care provided^b^	For chronic condition	218 (55.1)
	For acute condition	102 (25.8)
	Preventative	76 (19.2)

N, number of systematic reviews (unless otherwise indicated).

*Based on 358 systematic reviews unless otherwise specified.

†38 systematic reviews did not find published studies (n = 22) or include studies that had participants <18 (n = 16).

Based on 6,022 studies identified in 358 systematic reviews. Actual number of studies may be less, as some studies may be included in multiple systematic reviews.

‡Based on 353 systematic reviews, 5 systematic reviews were unclear or did not specify total number of participants.

§Based on 351 systematic reviews, 7 systematic reviews were unclear or did not specify total number of participants.

**Based on 394 systematic reviews, two systematic reviews were classified as ‘stable’.

††Based on 358 systematic reviews that identified studies. If a systematic review had greater than one category of intervention, it was double counted (n = 15); total interventions evaluated is 376.

b Based on all 396 systematic reviews.

A greater percentage of systematic reviews on complex interventions (n = 9, 24%) and natural health products (n = 8, 21%) found no relevant studies or children participants compared to included reviews (complex, n = 43, 11%; natural health product, n = 34, 9%). Of the 38 reviews that did not find any published studies, the main conditions studied were: respiratory (n = 10), blood and immune mechanism (n = 6), psychological (n = 6), digestive (n = 3) and neurological (n = 3).

The first systematic review was published in 1996 and since 2003, the rate of new publications has remained relatively constant at a median of 37 per year (range: 28–44). Nearly half of the systematic reviews (44%) were updated in 2008 or more recently, up-to-date as defined by the Cochrane Collaboration. Of reviews considered out-of-date, two were last updated in 1998.

**Figure 2:**
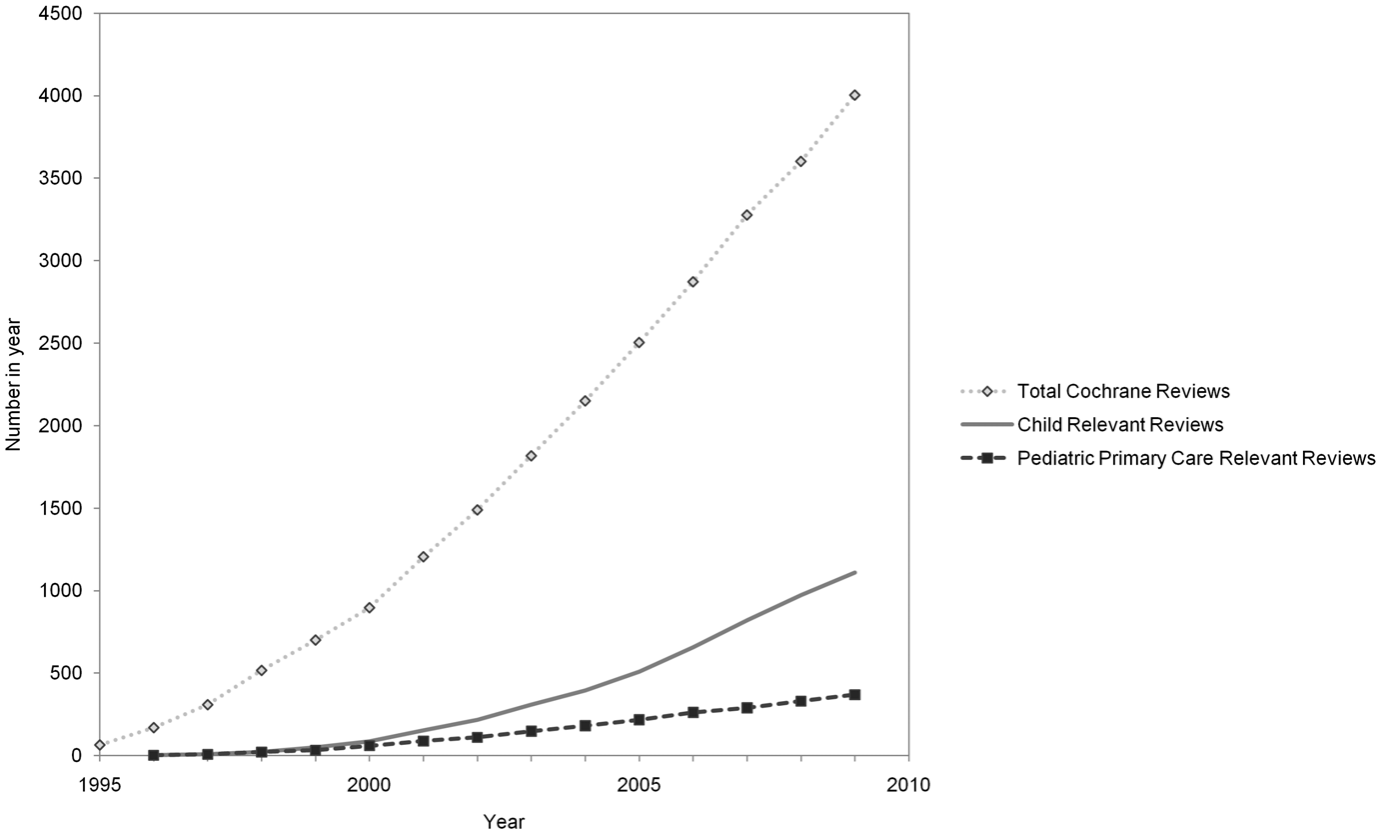
The cumulative number of systematic reviews published in the Cochrane Database of Systematic Reviews, 1995 to 2009.

Figure 2 illustrates the increase in the number of systematic reviews in the CDSR relevant to pediatric primary care compared to the increase in all child relevant and all Cochrane reviews. In 2009, there were 4,005 Cochrane reviews, of which 1,110 (28%) were on children and 370 (9%) were relevant to pediatric primary care. Since 2000, the percentage of child relevant reviews nearly tripled (from 10% to 28%) compared to a much smaller increase of 2% (from 7% to 9%) in pediatric primary care relevant reviews.

**Figure 3:**
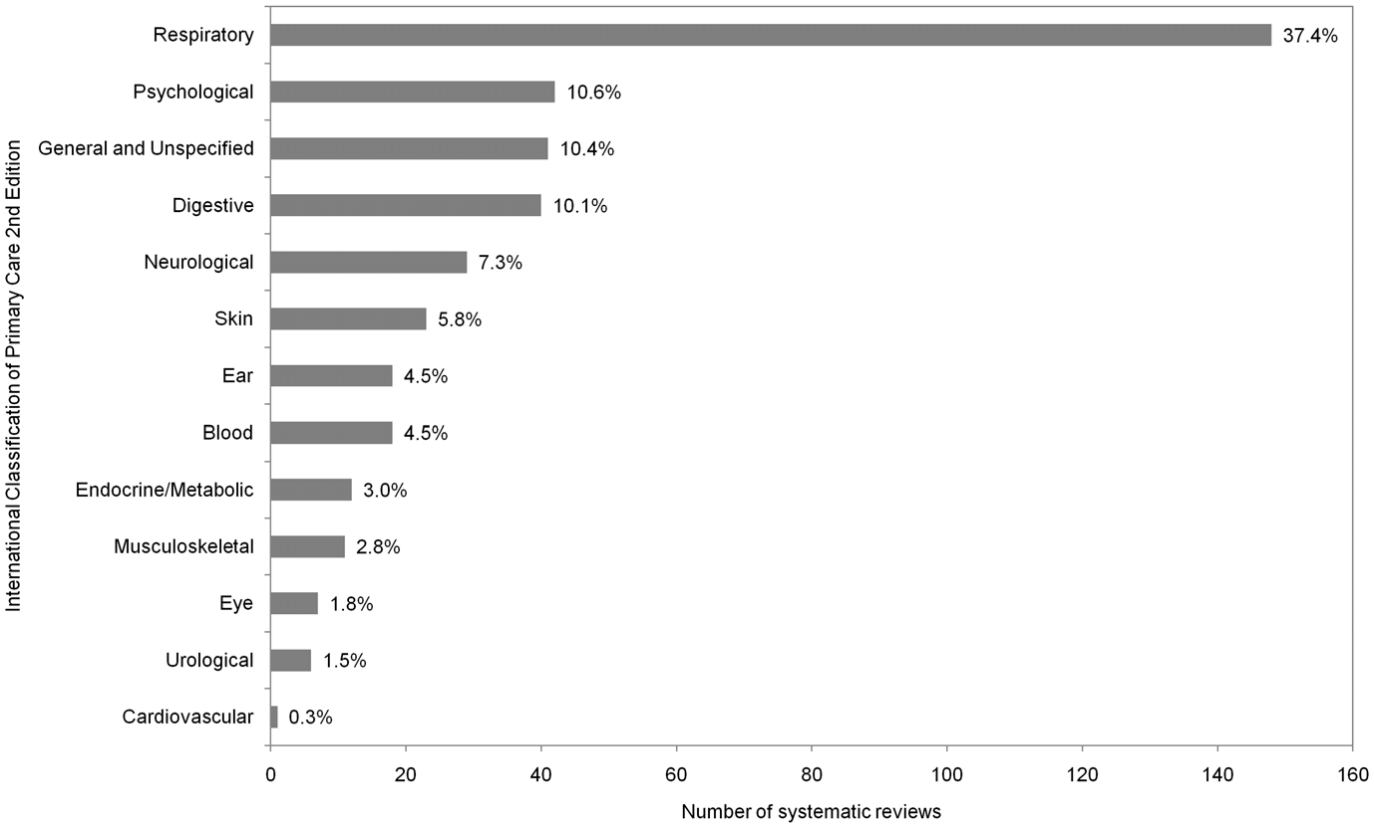
Classification of Cochrane systematic reviews relevant to children in primary care. All Cochrane systematic reviews relevant to children in primary care were classified according to the International Classification of Primary Care 2nd Edition.

Figure 3 illustrates systematic reviews categorized using the International Classification of Primary Care 2^nd^ Edition. Of note, five conditions cover 76% of all systematic reviews: respiratory (37%, n = 148), psychological (11%, n = 42), general and unspecified (e.g. fever; 10%, n = 41), digestive (10%, n = 40), and neurological (7%, n = 29).

### Consultations in primary care

Table 2 shows the most common reasons for childhood consultations in Australian and Dutch general practice, excluding systematic reviews relevant to developing countries only (n = 55). Respiratory conditions are the most common reason for general practice consultations in children representing 23–32% of all consultations, of which 43% (n = 147) of included systematic reviews are relevant. There was a discrepancy between the burden of illness and the corresponding number of systematic reviews. For instance, despite representing over 15% of general practice consultations, only 7% (n = 23) of systematic reviews were relevant to skin conditions. In contrast, 12% (n = 42) of systematic reviews were on psychological conditions even though these only represent a relatively small proportion of consultations in Australian and Dutch general practice (2–3%).

**Table 2:** Comparison of childhood consultations in Australian and Dutch general practice with number of systematic reviews.

	Cochrane systematic reviews*				
International Classification of Primary Care 2^nd^ Edition	N	%	Australia 2006–08, 0–15y (%)	Netherlands 2001, 0–17y (%)	Netherlands 1987, 0–17y (%)
Respiratory	147	43.1	31.5	23.3	25.5
Psychological	42	12.3	2.1	2.6	1.7
Digestive	28	8.2	7.5	8.1	8
Neurological	28	8.2	1.4	2.4	1.7
General and unspecified	23	6.7	19.9	7.8	15.6
Skin	23	6.7	15.3	23	17.8
Ear	17	5	8.9	8.9	7.6
Musculoskeletal	11	3.2	4.6	10	9.3
Endocrine/Metabolic	8	2.3	1.4	0.9	1.2
Urological	6	1.8	1.6	1.9	1.3
Eye	5	1.5	3.1	4.1	3.8
Blood	3	0.9	0.6	1	1.3
Cardiovascular	---	---	0.4	0.5	0.5
Pregnancy/labour	---	---	0.1	2.3	1.7
Female reproductive tract	---	---	0.7	1.8	1.3
Male reproductive tract	---	---	0.9	1	0.7
Social problems	---	---	0.2	0.4	0.6

N, number of systematic reviews.

*Based on 341 systematic reviews; excluded systematic reviews relevant to developing countries only (n = 55).

Table 3 evaluates specific diagnostic categories in greater detail and shows that despite asthma representing 3–5% of consultations in Australia, UK and US, it is the focus of 23% (n = 78) of systematic reviews. However, only 2% (n = 8) of systematic reviews were on injuries despite being the reason for 7–10% of consultations.

**Table 3:** Comparison of reasons for childhood consultations in Australia, UK and US with number of systematic reviews.

	Cochrane systematic reviews*				
Principal diagnosis category (ICD-9)	N	%	US 1993–95, 0–15y (%)	UK 2005–07, 0–15y (%)†	Australia 2000–01, 0–15y (%)‡
Asthma	78	22.9	2.5	3.3	4.6
Respiratory conditions§	62	18.2	22.7	23.9	21.8
Well-child visit	38	11.1	14.7	---	9.5
Ear conditions	16	4.7	13	9.1	7.7
Eczema	10	2.9	2	7.6	3
Injury	8	2.3	10.2	6.8	---
Gastroenteritis	7	2.1	0.4	1.9	1.4
Viral syndrome	6	1.8	2.3	8.3	---
Eye conditions	5	1.5	2.4	6.4	---
Bladder infection	5	1.5	0.6	0.8	---
All other	106	31.1	29	31.9	52

ICD-9, International Classification of Disease Revision 9; N, number of systematic reviews.

*Based on 341 systematic reviews; excluded systematic reviews relevant to developing countries only (n = 55).

†Weekly Returns Service data does not include ICD-9 ‘V-codes’ (relating to supplementary classification of factors influencing health status and contact with health services), therefore no UK data is available for well-child visit.

‡Limited Australian data available for comparison.

§Excluding asthma.

---Did not report data to stratify into diagnostic category.

### Major causes of under-five mortality

Table 4 compares the major causes of death in children younger than age 5 years in Africa and Southeast Asia and shows that while certain topics have a large proportion of systematic reviews, such as HIV/AIDS (22%, n = 12) and diarrheal diseases (22%, n = 12), childhood injuries and pneumonia have a relative lack of Cochrane reviews.

**Table 4:** Comparison of the major causes of death in children younger than age 5 years other than neonates in Africa and Southeast Asia with number of relevant systematic reviews.

	Mortality distribution		Distribution of Cochrane systematic reviews			
			Research mainly completed in low-income countries (N = 55)		Research completed in any country with possible relevance to low-income context (N = 63)	
Major causes of death§	Africa (%)	Southeast Asia (%)	N	%	Vaccine research, N = 14 (N)	Other research, N = 49 (N)
Pneumonia	28.8	34.5	3	5.5	4	13
Diarrheal diseases	21.9	32.7	12	21.8	2	7
Malaria	24.7	<1	17	30.9	---	---
Measles	6.8	5.5	1	1.8	1	2
Injuries	2.7	3.6	---	---	---	6
HIV/AIDS	8.2	1.8	12	21.8	---	---
Other	6.8	21.8	10	18.2	7*	21
Total number of deaths (million)	3.2	1.7	---	---	---	---

N, number of systematic reviews.

§Number of deaths in children younger than age 5 years other than neonates and their distribution by cause (yearly average for 2000–03) according to the World Health Organization Child Health Epidemiology Reference Group.

*Includes immunizations for Diphtheria-tetanus-pertussis (n = 1), Hepatitis A (n = 1), Hepatitis B (n = 1), N. meningitidis (n = 2), Varicella (n = 1), Patient reminder and recall systems (n = 1).

## Discussion

### Statement of principal findings

Our study is the first comprehensive mapping of the applicability of Cochrane systematic reviews to a large clinical topic – children in primary care – including approximately 10% of all reviews published in the Cochrane database. Although previous studies have shown that Cochrane systematic reviews only represent 20% of all published reviews [15], they are the principal international source of quality-assessed evidence on clinical effectiveness for health care workers and policy decision makers.

Our findings show some striking mismatches between the conditions for which the Cochrane database provides clinical evidence and the burden of childhood illness in primary care. For example, despite the increasing incidence of skin conditions (such as rash, eczema and impetigo) which precipitate up to 23% of all primary care consultations [21], [24], [29], only 7% of included Cochrane reviews are relevant to this topic. In contrast, 23% of reviews were on asthma which accounts for only 3–5% of clinical consultations. There are no similar primary care consultation data for low-income countries, but while the importance of HIV and malaria as important causes of childhood mortality is recognized in the Cochrane database, there is a relative lack of Cochrane review evidence on pneumonia and childhood injuries. Both conditions are commonly seen in primary care in low-income countries and are associated with high mortality [10].

### Possible explanations for the mismatch with burden of illness in primary care

There are several potential reasons for the observed discrepancy in the number of Cochrane reviews and the burden of consultations. First, systematic reviews are based on primary research and the deficiencies may reflect the lack of primary studies available for synthesis. For example, reviews of complex interventions and natural health products were more likely to identify studies with adult participants only or find no relevant studies. The lack of controlled trials in child health is well documented and due to numerous reasons [30]. Second, clinicians and researchers identify topics for Cochrane systematic reviews based on clinical uncertainty, practice variation or research interests. These may not correlate with the number of consultations or burden of illness. Third, public funding of research is correlated only modestly with disease burden, if at all [15]. Fourth, there may be more interventions (and comparisons) available for certain conditions than others. Fifth, certain areas may be broader in scope and represent more varied conditions (e.g. neurological disorders) that warrant a disproportionately greater volume of research relative to their consultation burden. Finally, the lack of pediatric training for general practitioners in many countries and therefore lack of academic interest in pediatric primary care is likely to contribute to the lack of research endeavor.

### Relevance to previous research

Previous studies have suggested that there may be insufficient evidence specific to children in certain topic areas [13], [31], [32], [33], especially non-drug interventions such as counseling and advice [34]. The emphasis on drug interventions is reflected in the funding of both commercial research and non-commercial research [35] while 69% of all pediatric controlled trials assess drug products [30]. In our study, we also found an over-representation of reviews relevant to drug interventions. Non-drug interventions are an important part of primary care and need to be reflected in evidence syntheses and research funding.

It is essential that systematic reviews address questions that are relevant to patients, clinicians and policy makers [14]. Several research groups have attempted to quantify the evidence that is available and used by physicians making treatment decisions [36], [37]. Even when reviews are applicable to children in general practice often authors fail to provide a description of how the intervention can be applied in clinical practice, reinforcing the findings of previous studies [38], [39]. Current work is being completed to determine how primary care practitioners use and apply Cochrane reviews in practice.

### Methodological limitations in the conduct of systematic reviews

Several of the systematic reviews in our study that used a meta-analysis included both children and adults; this observation is consistent with other studies [13], [16]. Combining studies with adults and children does not identify potentially important differences in efficacy and safety between these population groups [13] and can result in ineffective or even unsafe medical care [13], [40]. To derive relevant evidence for decision-making, systematic reviews should consider potentially important differences in effects across different populations. Guidance to inform subgroup analyses is currently being prepared through StaR Child Health (www.ifsrc.org), an initiative aimed at improving the design, conduct, and reporting of pediatric research [40].

Cochrane systematic reviews are expected to be updated every two years, [41] yet 56% of systematic reviews were last assessed as up-to-date prior to 2008 (as of August 2010), greater than 38% reported elsewhere [16]. Several authors have reiterated the importance of developing mechanisms and channeling resources to ensure that systematic reviews are updated as new evidence emerges [14], [42].

### Limitations of the study

Our definition of primary care setting included pediatric assessment units and emergency departments, which may have partially explained the discrepancy between consultations and reviews. However, we only included studies that could be administered in the primary care setting by a healthcare provider. Our analysis was restricted to Cochrane reviews of interventions; there may be systematic reviews published outside the CDSR that fill some of the clinical gaps identified, particularly screening, diagnostic and prognostic reviews. In addition, we did not assess the quality of included systematic reviews. Classification of the reviews by ICPC-2 and ICD-9 has several limitations and was completed to provide an approximate comparison rather than precise figures.

### Implications for research in children in primary care

The study highlights the on-going challenges of evidence based practice in primary care and the importance of characterizing the current evidence base. Further steps are required to identify and prioritize which systematic reviews are needed in primary care and encourage Cochrane Review Groups, funders, and other relevant organizations to promote those topics among potential authors. The recently implemented initiative to register all systematic review protocols is a step forward [43], [44]. There is a need for clinicians, researchers and policy makers in primary care to improve the evidence base in children by determining if the current evidence base adequately informs clinical practice.

## Supporting Information

Appendix S1 **Search terms used by the Cochrane Child Health Field.** The search strategy used to develop the Cochrane Child Health Field Reviews Register, a register of all child health relevant systematic reviews published in the CDSR.
(DOC)

Appendix S2 **Methodology of classifying systematic reviews by burden of illness data.**
(DOC)

Table S1 **List of all included Cochrane systematic reviews.**
(DOC)

PRISMA Checklist S1. (DOC)

PRISMA Flow Diagram S1. (DOC)
