# Supplementary material for: The Evidence Base for Interventions Delivered to Children in Primary Care: An Overview of Cochrane Systematic Reviews
Source: PLoS One. 2011 Aug 1;6(8):e23051. doi: 10.1371/journal.pone.0023051 (PMC3148227; doi:10.1371/journal.pone.0023051)
Supplement: Appendix S1 — Search terms used by the Cochrane Child Health Field. The search strategy used to develop the Cochrane Child Health Field Reviews Register, a register of all child health relevant systematic reviews published in the CDSR. (DOC) [file pone.0023051.s001.doc]

# Appendix S1. Search terms used by the Cochrane Child Health Field.

Cochrane Database of Systematic Reviews (Issue 2 2009)

Search conducted: 28 April 2009

ID Search

#1 (Infant* OR infancy OR Newborn* OR Baby* OR Babies OR Neonat* OR Preterm* OR Prematur* OR Postmatur* OR Child* OR Schoolchild* OR School age* OR Preschool* OR Kid or kids OR Toddler* OR Adoles* OR Teen* OR Boy* OR Girl* OR Minors* OR Pubert* OR Pubescen* OR Prepubescen* OR Pediatric* OR Paediatric* OR Peadiatric* OR Nursery school* OR Kindergar* OR Primary school* OR Secondary school* OR Elementary school* OR High school* OR Highschool*):ti,ab,kw in Cochrane Reviews

#2 "Types of Participants" NEAR/8 ("any age" or "children" OR "infants" OR "neonates" or "teenagers" or "adolescents" or "all ages" or "18 years or younger") in Cochrane Reviews

#3 (#2 OR #1)

**References**

Bow S, Klassen J, Chisholm A, Tjosvold L, Thomson D, et al. (2010) A descriptive analysis of child-relevant systematic reviews in the Cochrane Database of Systematic Reviews. BMC Pediatr 10: 34.
