# Supplementary material for: The Evidence Base for Interventions Delivered to Children in Primary Care: An Overview of Cochrane Systematic Reviews
Source: PLoS One. 2011 Aug 1;6(8):e23051. doi: 10.1371/journal.pone.0023051 (PMC3148227; doi:10.1371/journal.pone.0023051)
Supplement: Appendix S2 — Methodology of classifying systematic reviews by burden of illness data. (DOC) [file pone.0023051.s002.doc]

# Appendix S2. Methodology for comparison of burden of illness to systematic reviews.

## Introduction

The following supplementary file outlines the methodology behind comparing the burden of illness in general practice with the number of Cochrane systematic reviews relevant to children in primary care. The purpose of classifying the systematic reviews is not to provide precise estimates of burden of illness compared to number of reviews, but rather to give general estimates of how the number and scope of systematic reviews reflects the burden of illness in several developed countries.

Few countries publically report national burden of illness data in primary care. To our knowledge, only Australia and the United Kingdom regularly publish updated data on the burden of childhood illness in general practice [1,2]. In the United States, the Centers for Disease Control and Prevention and the National Center for Health Statistics publish reports from the National Ambulatory Medical Care Survey (NAMCS) and the National Hospital Ambulatory Medical Care Survey (NHAMSC) periodically with data from specific time periods [3]. Lastly, we obtained Dutch data on childhood morbidity in general practice through a literature search [4,5]. We compared the number of systematic reviews to the burden of childhood illness in general practice from these four countries.

## Coding system

The World Health Organization has developed a Family of International Classifications to promote the appropriate selection of classifications in the range of settings in the health field across the world [6]. There are two internationally used systems to classify diseases and other health problems recorded on many types of health and vital records in primary care: the International Classification of Diseases (ICD) and the International Classification of Primary Care Second Edition (ICPC-2) [7,8].

The ICD is the international standard diagnostic classification for all epidemiological, health management and clinical use, such as monitoring of the incidence and prevalence of diseases and analysis of the general health situation of population groups [7]. The ICPC-2, developed by the World Organization of Family Doctors, was specifically developed for primary care and classifies clinical activity in the domains of General/Family Practice, taking into account the frequency distribution of problems seen in these domains [8].

## Classification of systematic reviews

We classified all included systematic reviews by:

- Specific diagnosis as described in systematic review
- ICPC-2 chapter
- ICPC-2 chapter descriptor
- ICD-9 disease related group (3 digit code)

We classified the systematic reviews according to disease related groups as it provided an approximation of broad diagnostic groups. We used ICD-9 rather than ICD-10 as the datasets available used ICD-9. See Table 1 for an example of review classification.

**Table 1** Example of classification of Cochrane SRs

| **Systematic review title** | **Diagnosis** | **ICPC-2 Chapter (code)** | **ICPC-2 Chapter Component**  **(code)** | **ICD-9 disease related group**  **(code range)** |
| --- | --- | --- | --- | --- |
| Antibiotics for acute otitis media in children [9] | Otitis media | Ear (H) | Acute otitis media/myringitis (H7) | Ear and Mastoid Process diseases (380-389) |
| Antibiotics for the common cold and acute purulent rhinitis [10] | Respiratory tract infection | Respiratory (R) | Upper respiratory infection acute (R74) | Ear and Mastoid Process diseases (380-389) |

All the systematic reviews were first classified by ICD-9 disease related groups. Subsequently, they were classified by ICPC-2, and then checked a second time to verify that the ICD-9 disease related groups were consistent with the ICPC-2 classifications. One author (PG) classified reviews by diagnosis and it was checked by a second (KW). Subsequently, one author (PG) classified all reviews by ICD-9 and ICPC-2; uncertainty was resolved by consensus or consultation with a third author (AH or DM) at all stages.

The following decision rules were adhered to for ICPC-2 classification:

- If specific disease in the systematic review was not coded for in the ICPC-2, it was coded as the relevant chapter followed by “28” if it referred to a symptom complex (e.g. U28 = Intermittent catheterization for bladder management) or “99” if it referred to a disease (e.g. N99 = cerebral palsy).
- If a systematic review is about a vaccine targeted for an organism to prevent a disease outcome, it was coded as the chapter for the relevant infection it was preventing (e.g. N71 = meningitis) if it was different from the causative organism (e.g. A78 = meningococcal infection).
- If the systematic review was relevant to general health promotion/prevention, it was coded as “A98” for general and unspecified (e.g. growth monitoring).

Table 2 outlines a list of the ICPC-2 chapters for reference and Table 3 outlines the corresponding ICD-9 disease related groups and ICPC-2 chapter components.

**Table 2** List of ICPC-2 Chapters

| **ICPC-2 Chapters** | |  |  |
| --- | --- | --- | --- |
| A | General and unspecified | R | Respiratory |
| B | Blood and Immune Mechanism | S | Skin |
| D | Digestive | T | Endo/Metabolic and Nutritional |
| F | Eye | U | Urological |
| H | Ear | W | Pregnancy, Childbearing, Family Planning |
| K | Cardiovascular | X | Female reproductive tract |
| L | Musculoskeletal | Y | Male reproductive tract |
| N | Neurological | Z | Social problems |
| P | Psychological |  |  |

**Table 3** List of corresponding ICD-9 disease related groups and ICPC-2 chapter components

| **ICD-9 disease related group name** | **ICD-9 Code Range** | **ICPC-2 chapter components** |
| --- | --- | --- |
| Intestinal Infectious Diseases | 001~009 | D10, D11, D70, D73 |
| Viral Diseases Accompanied by Exanthema | 050~057 | A71, A72  S71 (Herpes simplex) |
| Viral Chlamydial Disease Other | 070~079 | A75, S95 |
| Mycoses | 110~118 | S74 |
| Infectious Parasitic Diseases Other | 130~136 | S72 |
| Malignant Neoplasm Lymphatic Haematological tissue | 200~208 | A29, A98 |
| Endocrine Gland Disease Other | 250~259 | T89, T90 |
| Nutritional Deficiencies | 260~269 | T91, T99 |
| Metabolic Immunity disorders Other | 270~279 | T82, T93 |
| Blood and Blood Forming Organs Diseases | 280~289 | B78, B80, B83 |
| Psychoses Other | 295~299 | P73, P76, P99 |
| Neurotic Disorders, Personality Disorders, and Other Nonpsychotic Mental Disorders | 300~316 | D17, L28, N19, P12, P19, P22, P24, P28, P74, P75, P76, P81, P82, P86, P99 |
| Central Nervous System Inflammatory Disease | 320~326 | N71 |
| Central Nervous System Disorders Other | 340~349 | N01, N88, N89, N95, N99 |
| Eye and Adnexa Disorders | 360~379 | F95, F99 |
| Ear and Mastoid Process Diseases | 380~389 | H04, H71, H72, H77, H81 |
| Acute Respiratory Infections | 460~466 | R29, R71, R72, R74, R75, R77, R78, R80, R81, A78 |
| Other Diseases of the Upper Respiratory Tract | 470~478 | R21, R90, R96, R97 |
| Chronic Obstructive Pulmonary Disease and Allied Conditions | 490~496 | R96 |
| Diseases of Oral Cavity, Salivary Glands, and Jaws | 520~529 | D82 |
| Oesophagus Stomach Duodenum Dis | 530~537 | D84 |
| Intestines and Peritoneum Disease Other | 560~569 | D12, D93, D95 |
| Urinary System Diseases Other | 590~599 | U70, U71, U99 |
| Infections of Skin and Subcutaneous Tissue | 680~686 | S76, S84 |
| Other Inflammatory Conditions of Skin and Subcutaneous Tissue | 690~698 | S87, S89, S91 |
| Arthropathies and Related Disorders | 710~719 | L17, L88, L99 |
| Osteopathies, Chondropathies, and Acquired Musculoskeletal Deformities | 730~739 | L95, L98 |
| Symptoms and Signs Ill-defined | 780~789 | A03, D01, R03, R04, R05, R06 |
| Dislocations | 830~839 | L10 |
| Sprains and Strains of Joints and Adjacent Muscles | 840~848 | L19 |
| Open Wound Head Neck and Trunk | 870~879 | S13, S18, S19 |
| Burns | 940~949 | S14 |
| Other and Unspecified Effects of External Causes | 990~995 | A88 |
| V-code | V-code | A72, A73, A98, D70, D72, H71, L28, N71, P17, P28, P99, R80, R81, R99, U28 |

## Consultation burden

*Australia*

The Bettering the Evaluation and Care of Health (BEACH) Project collects data from around 1,000 general practitioners, who each record details about 100 consecutive consultations, thus providing an annual database of approximately 100,000 records [11]. By randomly selecting general practices from across Australia, they provide good estimates of national general practice encounters.

We used publically available data from the BEACH Project and the Australian Institute of Health and Welfare (AIHW) to determine the burden of illness of children in general practice [12]. The BEACH Project reports data as the percentage of ICPC-2 problem chapters managed at general practice encounters for patients in <1, 1-4 and 5-14 years old but does not provide population figures. However, the BEACH Project publishes annual general practice activity reports for the AIHW, therefore we assumed that the number of patients in each age group published in the AIHW reports is consistent with the denominator used to generate the estimates provided on the BEACH website.

We collected data for burden of illness for the financial years 2005-06, 2006-07 and 2007-08 from the BEACH website and total number of patients in <1, 1-4 and 5-14 age groups from the AIHW general practice activity reports. Next, we derived the percentage distribution of problem chapters for all children under 15 years and calculated the percentage of encounters for each ICPC-2 chapter for all patients under 15 years. The percentage burden of each ICPC-2 chapter was calculated using the ratio of the number of encounters in the ICPC-2 chapter to the total number of consultations in one year in children under 15. To reduce year-to-year fluctuations in data, we analyzed three years of data (2005-07). We report the average percentage burden over three years for the comparison. Furthermore, we extracted the data available from an additional peer-reviewed study on Australian general practice data directly into a table for comparison [4].

*Netherlands*

We identified a published report evaluating the changing morbidity patterns in children in Dutch general practice comparing 1987 and 2001 [5]. The data presented in the study is from the second Dutch national survey of morbidity and interventions, which was performed by the Netherlands Institute for Health Services Research in 2001, in addition to data from the first Dutch national survey completed in 1987. We extracted the data available directly into a table for comparison.

*United Kingdom*

The Royal College of General Practitioners Weekly Returns Service (WRS) is a sentinel general practice network located across England and Wales that consists of a nationally representative population [2,13,14]. The WRS routinely monitors all disease episodes from every general practice consultation on a weekly basis and classifies them according to the ICD-9. Participating practices provide information on the number of patients seen each week categorized by gender, age group and disease group along with the detailed age specific practice registered population data. If no clear diagnosis can be made, practices enter the predominant symptom code as a diagnostic Read code to facilitate mapping to the ICD-9 Chapter Symptoms, Signs and Ill-defined conditions. The WRS annual prevalence data was available on a yearly basis until 2007. The WRS included 91 practices in 2005, 47 practices in 2006 and 64 practices in 2007. The reports present the age and gender specific data for all persons and gender organized by ICD-9 chapter, disease related groups and by first 3 ICD digits [2].

Using the WRS, we determined the number of consultations of each ICD-9 disease related group using the patient consulting rate per 10,000 persons and population of each age group under 15 (<1, 1-4 and 5-14) reported in the WRS. Denominator data comprised of the total number of consultations for all disease related groups over a one-year period for children under 15 (<1, 1-4 and 5-14). To calculate the numerator, we combined the number of consultations of each disease related group for children under 15 over a one-year period. The percentage burden of each disease related groups was calculated using the ratio of the number of consultations in the disease related group to the total number of consultations in one year in children under 15. In order to reduce the possibility of year-to-year fluctuations in the data, we analyzed three years of data (2005-07). The final value used for the percentage burden in general practice was the average value over the three years.

*United States*

Burden of illness data was obtained from the NAMCS and the NHAMCS [3]. Both national surveys are designed to collect objective, reliable information about the provision and use of ambulatory medical care services (NAMCS) and hospital emergency and outpatient departments (NHAMCS) in the United States [3]. Specially trained interviewers visited the physicians before their participation in the survey to instruct them on how to complete the forms and provide them with survey and reference materials. Selected physicians completed questionnaires for a systematic random sample of all patient visits made during one week. NAMCS findings are based on a sample of included visits made to non–federally employed, office-based physicians in the United States. NHAMCS findings are based on a national sample of visits to the emergency departments and outpatient departments of non-institutional general and short-stay hospitals. Additional details of the survey’s methods are available elsewhere [3].

United States data on the burden of illness in ambulatory care by principal diagnosis in children under 15 was generated from the NAMCS and NHAMCS Survey. The physician’s principal diagnosis, associated with the patient’s stated reason for this visit, was collected and coded to the ICD-9 Clinical Modification (ICD-9-CM). The most recent comprehensive data that could be determined by principle diagnosis were from 1993-95 [3]. The average annual 1993-95 percent distribution of the ambulatory care visits among children under 15 years of age by place of visit and principal diagnosis category was reported for 20 clinically significant pediatric diagnosis categories. Information was not provided on all ICD-9-CM categories. A list of the principal diagnoses and corresponding ICD-9-CM codes is attached in Appendix 1. Additional data on the rate of ambulatory care visits for select conditions was identified for 2001-02,[15] however we did not include it in the comparison table as it was only relevant to select conditions and did not contribute to the comparison.

## Summary tables of burden of illness

Subsequently, we compared the number of systematic reviews and the burden of illness in general practice. Systematic reviews primarily relevant to developing countries only (n=55) were excluded from the comparison, as the burden of illness data is from developed countries only. Therefore, 341 systematic reviews were included in the comparison.

Using ICPC-2 chapters, we compared the burden of illness in Australia and the Netherlands to the number of systematic reviews. Due to the organization of the ICD-9 by diagnosis, there were difficulties in correlating it with the ICPC-2 system, which is organized by symptom. Therefore, we created two tables, one for ICPC-2 (Australia and Netherlands) and one for ICD-9 (US, UK and select additional Australian data).

Due to the lack of complete data available reported in the US (only 20 diagnosis categories reported in detail, see Table 4 and Appendix 1), we were unable to compare all ICD-9 codes with UK data. However, we used ten principle diagnoses categories outlined in the NAMCS and NHAMCS Survey report to compare US with UK and Australian data. We matched ICD-9 disease related groups to the specific principle diagnosis categories used in the US, including to an additional section with Australian data reported in the literature (Table 4) [4].

**Table 4** List of ICD-9CM codes

| **Ambulatory health care visits by children: principal diagnosis** | | **ICD-9 Disease Related Group** | **Australia 2000-01[4]** |
| --- | --- | --- | --- |
| **Principle diagnosis category** | **Corresponding ICD-9 Code** |
| **Well-child visit** | V20.2, V70.0, V70.5, V70.9 | Not collected in the UK WRS | Vaccination |
| **Injury** | 800–999 | All DRG groups 800-999 | N/A |
| **Select respiratory conditions** | | 460-466 (Acute respiratory infections)  470-478 (Other upper respiratory tract diseases)  480-487 (Pneumonia and Influenza) | Upper respiratory tract infection  Tonsillitis  Bronchitis/bronchiolitis |
| *Cold, cough, runny nose* | 460.0, 464.0, 465.9, 472.0, 786.2. |
| *Sore throat, tonsillitis* | 462.0, 463.0, 472.1–472.2, 474, 475 |
| *Hayfever, sinusitis* | 461, 473, 477 |
| *Bronchitis* | 466.0, 490, 491 |
| *Pneumonia* | 466.1, 480, 481, 482, 483, 484, 485, 486, 487.0 |
| *Streptococcal sore throat* | 034.0, 034.1 |
| *Croup* | 464.1–464.4 |
| **Asthma**† | 493 | 490-496 (Pulmonary Dis Chronic Obst & Allied cond) | Asthma |
| **Select ear conditions** | | 380-389 (Ear and Mastoid Process diseases) | Otitis media  Otitis externa |
| *Middle ear infection* | 381.0–381.4, 382 |
| *External ear canal infection* | 380.1–380.16, 380.2–380.23 |
| **Select eye conditions** | | 360-379 (Eye and Adnexa Disorders) | N/A |
| *Infectious conjunctivitis* | 077.9, 372.0–372.05, 372.1–372.14, 372.30, 372.39, 372.9 |
| *Correctable vision* | 367.0, 367.1, 367.2, 367.3, 367.4, 367.5, 367.8, 367.9, 378.0–378.9 |
| **Viral syndrome** | 054.2, 054.9, 057.0, 057.8, 057.9, 074.0, 074.3, 074.8, 075.0, 079.0, 079.1, 079.2, 079.3, 079.8, 079.9, 487.1, 780.6 | 050-057 (Viral diseases with Exanthema)  070-079 (Viral Chlamydial Disease Other) | N/A |
| **Eczema** | 691, 692, 693, 708 | 690-698 (Skin & Subcutaneous Tiss Oth)  700-709 (Inflam Cond Skin & Subcutaneous Tiss Disease Other) | Eczema/dermatitis |
| **Bladder infection** | 595.0, 595.9, 599.0 | 590-599 (Urinary System Diseases Other) | N/A |
| **Gastroenteritis** | 008.6, 008.8, 009.0, 009.1, 009.2, 009.3 | 001-009 (Intestinal Infectious Diseases) | Gastroenteritis/dysentery |
| **All other diagnoses** | Includes all other diagnoses not mentioned | All other ICD-9 disease related groups  780-789 (Symptoms Signs and Illdefined) | Laceration  All other conditions |
| *Attention deficit disorder* | 314.0, 314.01 |
| *Chicken pox* | 052 |
|  |  |  |  |

Due to the large number of systematic reviews on asthma, we excluded it from the *Select respiratory conditions* category to compare it separately. We did not add the disease related group “700-709 Inflammatory Conditions Skin & Subcutaneous Tissue Disease Other” to *Eczema* as it only pertained to a single ICD-9 code (708). This group is included in the *All other diagnoses* section. Further, due to the size of “780-789 Symptoms Signs and Ill defined” disease related group in the UK (9.5%), we felt that as it related only to 786.2 it would be more appropriate to include this group in the *All other diagnoses* section rather than it remain in the *Select Respiratory Conditions* category.

As the RCGP WRS does not collect prevalence data on ICD-9 V-codes (relating to supplementary classification of factors influencing health status and contact with health services), systematic reviews that were labeled as V-codes and others classified as an ICD-9 code not listed in WRS were included in the *Well-child visit* category as they primarily were pertaining to health promotion.

## Limitations

We understand that there are several limitations in this method, which are inherently due to the lack of national collection of data by countries worldwide. We intended to classify the reviews by the ICD-9 code that most closely resembled the relevant condition while compromising on the complexity and level of detail required. We felt that the ICD-9 chapters would be too general due to the nature of classification. Therefore, disease related groups seemed to be the most reasonable compromise. However, due to the organization of the ICD-9 by diagnosis, there were difficulties in correlating it with the ICPC-2 system, which is organized by symptom. We have attempted to be as accurate as possible and to preserve the objective of the activity in coding.

## Appendix 1

List of all ICD-9 codes and descriptors

**Principal Diagnosis**

**Category and Population**

**Tables**

**Table I. Principal diagnosis categories and ICD–9–CM codes**

Principal diagnosis category Definition of ICD–9–CM code

Well-child visit

V20.2 . . . . . . . . . . . . . . . . . Routine infant or child health check

V70.0 . . . . . . . . . . . . . . . . . Routine general medical exam

V70.5 . . . . . . . . . . . . . . . . . Health exam of defined subpopulations

V70.9 . . . . . . . . . . . . . . . . . Unspecified general medical exam

Injury

800–999 . . . . . . . . . . . . . . . . Injury and poisoning

Cold, cough, runny nose

460.0 . . . . . . . . . . . . . . . . . . Acute nasopharyngitis

464.0 . . . . . . . . . . . . . . . . . . Acute laryngitis

465.9 . . . . . . . . . . . . . . . . . . Acute upper respiratory infection, unspecified site

472.0 . . . . . . . . . . . . . . . . . . Chronic rhinitis

786.2 . . . . . . . . . . . . . . . . . . Cough

Sore throat, tonsillitis

462.0 . . . . . . . . . . . . . . . . . . Acute pharyngitis

463.0 . . . . . . . . . . . . . . . . . . Acute tonsillitis

472.1–472.2 . . . . . . . . . . . . . Chronic pharyngitis/nasopharyngitis

474 . . . . . . . . . . . . . . . . . . . Chronic diseases of tonsils and adenoids

475 . . . . . . . . . . . . . . . . . . . Peritonsillar abscess

Hayfever, sinusitis

461 . . . . . . . . . . . . . . . . . . . Acute sinusitis

473 . . . . . . . . . . . . . . . . . . . Chronic sinusitis

477 . . . . . . . . . . . . . . . . . . . Allergic rhinitis

Bronchitis

466.0 . . . . . . . . . . . . . . . . . . Acute bronchitis

490 . . . . . . . . . . . . . . . . . . . Bronchitis, not specified as acute or chronic

491 . . . . . . . . . . . . . . . . . . . Chronic bronchitis

Asthma

493 . . . . . . . . . . . . . . . . . . . Asthma

Pneumonia

466.1 . . . . . . . . . . . . . . . . . . Acute bronchiolitis

480 . . . . . . . . . . . . . . . . . . . Viral pneumonia

481 . . . . . . . . . . . . . . . . . . . Pneumococcal pneumonia

482 . . . . . . . . . . . . . . . . . . . Other bacterial pneumonia

483 . . . . . . . . . . . . . . . . . . . Pneumonia due to specified organism

484 . . . . . . . . . . . . . . . . . . . Pneumonia in infectious disease

485 . . . . . . . . . . . . . . . . . . . Bronchopneumonia, organism unspecified

486 . . . . . . . . . . . . . . . . . . . Pneumonia, organism unspecified

487.0 . . . . . . . . . . . . . . . . . . Influenza with pneumonia

Streptococcal sore throat

034.0 . . . . . . . . . . . . . . . . . . Streptococcal sore throat

034.1 . . . . . . . . . . . . . . . . . . Scarlet fever

Croup

464.1–464.4 . . . . . . . . . . . . . Acute tracheitis, laryngotracheitis, epiglottitis, and croup

Middle ear infection

381.0–381.4 . . . . . . . . . . . . . Nonsupprative otitis media

382 . . . . . . . . . . . . . . . . . . . Supprative and unspecified otitis media

External ear canal infection

380.1–380.16 . . . . . . . . . . . . Infective otitis externa

380.2–380.23 . . . . . . . . . . . . Other otitis externa

Infectious conjunctivitis

077.9 . . . . . . . . . . . . . . . . . . Viral conjunctivitis, not otherwise specified

372.0–372.05 . . . . . . . . . . . . Acute conjunctivitis

372.1–372.14 . . . . . . . . . . . . Chronic conjunctivitis

372.30 . . . . . . . . . . . . . . . . . Conjunctivitis, unspecified

372.39 . . . . . . . . . . . . . . . . . Other conjunctivitis

372.9 . . . . . . . . . . . . . . . . . . Unspecified disorder of conjunctiva

Correctable vision

367.0 . . . . . . . . . . . . . . . . . . Hypermetropia

367.1 . . . . . . . . . . . . . . . . . . Myopia

367.2 . . . . . . . . . . . . . . . . . . Astigmatism

367.3 . . . . . . . . . . . . . . . . . . Anisometropia and aniseikonia

367.4 . . . . . . . . . . . . . . . . . . Presbyopia

367.5 . . . . . . . . . . . . . . . . . . Disorders of accomodation

367.8 . . . . . . . . . . . . . . . . . . Other disorders of refraction and accomodation

367.9 . . . . . . . . . . . . . . . . . . Unspecified disorder of refraction and accomodation

378.0–378.9 . . . . . . . . . . . . . Strabismus and other disorders of binocular eye movements

Viral syndrome

054.2 . . . . . . . . . . . . . . . . . . Herpetic gingivostomatitis

054.9 . . . . . . . . . . . . . . . . . . Herpes simplex without mention of complication

057.0 . . . . . . . . . . . . . . . . . . Erythema infectiosum

057.8 . . . . . . . . . . . . . . . . . . Other viral exanthemata (Fourth disease, roseola etc).

057.9 . . . . . . . . . . . . . . . . . . Viral exanthem, unspecified

074.0 . . . . . . . . . . . . . . . . . . Herpangina

074.3 . . . . . . . . . . . . . . . . . . Hand, foot, and mouth disease

074.8 . . . . . . . . . . . . . . . . . . Other diseases due to Coxsackie virus

075.0 . . . . . . . . . . . . . . . . . . Infectious mononucleosis

079.0 . . . . . . . . . . . . . . . . . . Adenovirus

079.1 . . . . . . . . . . . . . . . . . . ECHO virus

079.2 . . . . . . . . . . . . . . . . . . Coxsackie virus

079.3 . . . . . . . . . . . . . . . . . . Rhinovirus

079.8 . . . . . . . . . . . . . . . . . . Other specified viral infections

079.9 . . . . . . . . . . . . . . . . . . Viral infection, not otherwise specified

487.1 . . . . . . . . . . . . . . . . . . Influenza with other respiratory manifestations

780.6 . . . . . . . . . . . . . . . . . . Pyrexia of unknown origin

Eczema

691 . . . . . . . . . . . . . . . . . . . Atopic dermatitis and related conditions

692 . . . . . . . . . . . . . . . . . . . Contact dermatitis and other eczema

693 . . . . . . . . . . . . . . . . . . . Dermatitis due to substances taken internally

708 . . . . . . . . . . . . . . . . . . . Urticaria

Attention deficit disorder

314.0 . . . . . . . . . . . . . . . . . . Without hyperactivity

314.01 . . . . . . . . . . . . . . . . . With hyperactivity

Bladder infection

595.0 . . . . . . . . . . . . . . . . . . Acute cystitis

595.9 . . . . . . . . . . . . . . . . . . Cystitis, unspecified

599.0 . . . . . . . . . . . . . . . . . . Urinary tract infection, site not specified

Gastroenteritis

008.6 . . . . . . . . . . . . . . . . . . Enteritis due to specified virus

008.8 . . . . . . . . . . . . . . . . . . Other organism, not elsewhere classified

009.0 . . . . . . . . . . . . . . . . . . Infectious colitis, enteritis, and gastroenteritis

009.1 . . . . . . . . . . . . . . . . . . Gastroenteritis of presumed infectious origin

009.2 . . . . . . . . . . . . . . . . . . Infectious diarrhea

009.3 . . . . . . . . . . . . . . . . . . Diarrhea of presumed infectious origin

Chicken pox

052 . . . . . . . . . . . . . . . . . . . Varicella, with and without complications

All other diagnoses . . . . . . . . . Includes all other diagnoses not mentioned in
