# Supplementary material for: The Evidence Base for Interventions Delivered to Children in Primary Care: An Overview of Cochrane Systematic Reviews
Source: PLoS One. 2011 Aug 1;6(8):e23051. doi: 10.1371/journal.pone.0023051 (PMC3148227; doi:10.1371/journal.pone.0023051)
Supplement: Table S1 — List of all included Cochrane systematic reviews. (DOC) [file pone.0023051.s003.doc]

**Table S1.** List of all included Cochrane systematic reviews.

| **No.** | **Study** |
| --- | --- |
| 1 | McCarney RW; Brinkhaus B; Lasserson TJ; Linde K. Acupuncture for chronic asthma. Cochrane Database of Systematic Reviews. 2009, Issue 3. Art. No.: CD000008 |
| 2 | Cates CJ; Crilly JA; Rowe BH. Holding chambers (spacers) versus nebulisers for beta-agonist treatment of acute asthma. Cochrane Database of Systematic Reviews. 2009, Issue 1. Art. No.: CD000052 |
| 3 | Plotnick L; Ducharme F. Combined inhaled anticholinergics and beta2-agonists for initial treatment of acute asthma in children. Cochrane Database of Systematic Reviews. 2008, Issue 4. Art. No.: CD000060 |
| 4 | Yorke J; Shuldham C. Family therapy for asthma in children. Cochrane Database of Systematic Reviews. 2009, Issue 3. Art. No.: CD000089 |
| 5 | Rowe BH; Spooner C; Ducharme F; Bretzlaff J; Bota G. Corticosteroids for preventing relapse following acute exacerbations of asthma. Cochrane Database of Systematic Reviews. 2008, Issue 4. Art. No.: CD000195 |
| 6 | Sanders S; Glasziou PP; Del MC; Rovers M. Antibiotics for acute otitis media in children. Cochrane Database of Systematic Reviews. 2010, Issue 1. Art. No.: CD000219 |
| 7 | Arroll B; Kenealy T. Antibiotics for the common cold and acute purulent rhinitis. Cochrane Database of Systematic Reviews. 2010, Issue 2. Art. No.: CD000247 |
| 8 | McIntosh H; Olliaro P. Artemisinin derivatives for treating uncomplicated malaria. Cochrane Database of Systematic Reviews. 2010, Issue 1. Art. No.: CD000256 |
| 9 | Strong M; Johnstone P. Interventions for treating scabies. Cochrane Database of Systematic Reviews. 2009, Issue 1. Art. No.: CD000320 |
| 10 | Wolf F; Guevara JP; Grum CM; Clark NM; Cates CJ. Educational interventions for asthma in children. Cochrane Database of Systematic Reviews. 2008, Issue 4. Art. No.: CD000326 |
| 11 | Lengeler C. Insecticide-treated bed nets and curtains for preventing malaria. Cochrane Database of Systematic Reviews. 2009, Issue 2. Art. No.: CD000363 |
| 12 | Cates CJ; Jefferson T; Rowe BH. Vaccines for preventing influenza in people with asthma. Cochrane Database of Systematic Reviews. 2009, Issue 2. Art. No.: CD000364 |
| 13 | Taylor-Robinson DC; Jones AP; Garner P. Deworming drugs for treating soil-transmitted intestinal worms in children: effects on growth and school performance. Cochrane Database of Systematic Reviews. 2008, Issue 3. Art. No.: CD000371 |
| 14 | McIntosh H; Jones KL. Chloroquine or amodiaquine combined with sulfadoxine-pyrimethamine for treating uncomplicated malaria. Cochrane Database of Systematic Reviews. 2010, Issue 1. Art. No.: CD000386 |
| 15 | Ardern K. Dietary salt reduction or exclusion for allergic asthma. Cochrane Database of Systematic Reviews. 2008, Issue 4. Art. No.: CD000436 |
| 16 | Saconato H; Atallah ┴N. Interventions for treating schistosomiasis mansoni. Cochrane Database of Systematic Reviews. 2009, Issue 1. Art. No.: CD000528 |
| 17 | Linde K; Barrett B; Bauer R; Melchart D; Woelkart K. Echinacea for preventing and treating the common cold. Cochrane Database of Systematic Reviews. 2009, Issue 4. Art. No.: CD000530 |
| 18 | Graves PM; Deeks JJ; Demicheli V; Pratt M; Jefferson T. Vaccines for preventing cholera. Cochrane Database of Systematic Reviews. 2010, Issue 8. Art. No.: CD000974 |
| 19 | Hemil H; Chalker E; Treacy B; Douglas B. Vitamin C for preventing and treating the common cold. Cochrane Database of Systematic Reviews. 2010, Issue 3. Art. No.: CD000980 |
| 20 | Kaur B; Rowe BH; Arnold E. Vitamin C supplementation for asthma. Cochrane Database of Systematic Reviews. 2009, Issue 2. Art. No.: CD000993 |
| 21 | Hondras MA; Linde K; Jones AP. Manual therapy for asthma. Cochrane Database of Systematic Reviews. 2009, Issue 3. Art. No.: CD001002 |
| 22 | Marson AG; Williamson PR; Hutton JL; Clough HE; Chadwick DW. Carbamazepine versus valproate monotherapy for epilepsy. Cochrane Database of Systematic Reviews. 2009, Issue 4. Art. No.: CD001030 |
| 23 | Gamble CL; Williamson PR; Marson AG. Lamotrigine versus carbamazepine monotherapy for epilepsy. Cochrane Database of Systematic Reviews. 2009, Issue 4. Art. No.: CD001031 |
| 24 | Patel M; Lee Ck. Polysaccharide vaccines for preventing serogroup meningococcal meningitis. Cochrane Database of Systematic Reviews. 2010, Issue 8. Art. No.: CD001093 |
| 25 | Kozyrskyj A; Hildes-Ripstein GE; Longstaffe SE; Wincott JL; Sitar DS; Klassen TP; Moffatt M. Short course antibiotics for acute otitis media. Cochrane Database of Systematic Reviews. 2010, Issue 7. Art. No.: CD001095 |
| 26 | McKean MC; Ducharme F. Inhaled steroids for episodic viral wheeze of childhood. Cochrane Database of Systematic Reviews. 2009, Issue 1. Art. No.: CD001107 |
| 27 | Sirinavin S; Garner P. Antibiotics for treating salmonella gut infections. Cochrane Database of Systematic Reviews. 2009, Issue 1. Art. No.: CD001167 |
| 28 | Spooner C; Saunders LD; Rowe BH. Nedocromil sodium for preventing exercise-induced bronchoconstriction. Cochrane Database of Systematic Reviews. 2009, Issue 3. Art. No.: CD001183 |
| 29 | Stein DJ; Ipser JC; van-Balkom AJ. Pharmacotherapy for social anxiety disorder. Cochrane Database of Systematic Reviews. 2009, Issue 1. Art. No.: CD001206 |
| 30 | Chalmers R; O'Sullivan T; Owen CM; Griffiths-Christopher EM. Interventions for guttate psoriasis. Cochrane Database of Systematic Reviews. 2009, Issue 1. Art. No.: CD001213 |
| 31 | Fraser A; Goldberg E; Acosta CJ; Paul M; Leibovici L. Vaccines for preventing typhoid fever. Cochrane Database of Systematic Reviews. 2009, Issue 1. Art. No.: CD001261 |
| 32 | Gadomski AM; Bhasale AL. Bronchodilators for bronchiolitis. Cochrane Database of Systematic Reviews. 2009, Issue 1. Art. No.: CD001266 |
| 33 | Everard M; Bara A; Kurian M; N'Diaye T; Ducharme F; Mayowe V. Anticholinergic drugs for wheeze in children under the age of two years. Cochrane Database of Systematic Reviews. 2009, Issue 1. Art. No.: CD001279 |
| 34 | Tee A; Koh MS; Gibson PG; Lasserson TJ; Wilson A; Irving LB. Long-acting beta2-agonists versus theophylline for maintenance treatment of asthma. Cochrane Database of Systematic Reviews. 2009, Issue 3. Art. No.: CD001281 |
| 35 | Sharek PJ; Bergman D; Ducharme F. Beclomethasone for asthma in children: effects on linear growth. Cochrane Database of Systematic Reviews. 2008, Issue 4. Art. No.: CD001282 |
| 36 | Thien-Francis CK; De LS; Woods RK; Abramson MJ. Dietary marine fatty acids (fish oil) for asthma in adults and children. Cochrane Database of Systematic Reviews. 2010, Issue 7. Art. No.: CD001283 |
| 37 | Walters EH; Walters-Julia AE; Gibson PG; Jones P. Inhaled short acting beta2-agonist use in chronic asthma: regular versus as needed treatment. Cochrane Database of Systematic Reviews. 2009, Issue 3. Art. No.: CD001285 |
| 38 | Boyd M; Lasserson TJ; McKean MC; Gibson PG; Ducharme FM; Haby M. Interventions for educating children who are at risk of asthma-related emergency department attendance. Cochrane Database of Systematic Reviews. 2010, Issue 1. Art. No.: CD001290 |
| 39 | Jepson RG; Mihaljevic L; Craig JC. Cranberries for treating urinary tract infections. Cochrane Database of Systematic Reviews. 2009, Issue 4. Art. No.: CD001322 |
| 40 | Bassler D; Mitra-Andrew AD; Ducharme F; Forster J; Schwarzer G. Ketotifen alone or as additional medication for long-term control of asthma and wheeze in children. Cochrane Database of Systematic Reviews. 2010, Issue 7. Art. No.: CD001384 |
| 41 | Walters EH; Gibson PG; Lasserson TJ; Walters-Julia AE. Long-acting beta2-agonists for chronic asthma in adults and children where background therapy contains varied or no inhaled corticosteroid. Cochrane Database of Systematic Reviews. 2008, Issue 4. Art. No.: CD001385 |
| 42 | Panpanich R; Garner P. Growth monitoring in children. Cochrane Database of Systematic Reviews. 2009, Issue 1. Art. No.: CD001443 |
| 43 | Martins S; Logan S; Gilbert RE. Iron therapy for improving psychomotor development and cognitive function in children under the age of three with iron deficiency anaemia. Cochrane Database of Systematic Reviews. 2009, Issue 1. Art. No.: CD001444 |
| 44 | Kabra SK; Lodha R; Hilton DJ. Antibiotics for preventing complications in children with measles. Cochrane Database of Systematic Reviews. 2010, Issue 2. Art. No.: CD001477 |
| 45 | Yang HM; Mao M; Wan CM. Vitamin A for treating measles in children. Cochrane Database of Systematic Reviews. 2009, Issue 3. Art. No.: CD001479 |
| 46 | Jansen-Angelique GSC; Hak E; Veenhoven RH; moiseaux-Roger AMJ; Schilder-Anne GM; Sanders-Elisabeth AM. Pneumococcal conjugate vaccines for preventing otitis media. Cochrane Database of Systematic Reviews. 2009, Issue 2. Art. No.: CD001480 |
| 47 | Cates CJ; Bestall JC; Adams NP. Holding chambers versus nebulisers for inhaled steroids in chronic asthma. Cochrane Database of Systematic Reviews. 2009, Issue 3. Art. No.: CD001491 |
| 48 | Gibson PG; Henry R; Coughlan-Jennifer JL. Gastro-oesophageal reflux treatment for asthma in adults and children. Cochrane Database of Systematic Reviews. 2009, Issue 1. Art. No.: CD001496 |
| 49 | Hodson EM; Wheeler DM; Smith GH; Craig JC; Vimalachandra D. Interventions for primary vesicoureteric reflux. Cochrane Database of Systematic Reviews. 2009, Issue 3. Art. No.: CD001532 |
| 50 | Williams G; Wei L; Lee A; Craig JC. Long-term antibiotics for preventing recurrent urinary tract infection in children. Cochrane Database of Systematic Reviews. 2009, Issue 1. Art. No.: CD001534 |
| 51 | Pitkethly M; MacGillivray S; Ryan R. Recordings or summaries of consultations for people with cancer. Cochrane Database of Systematic Reviews. 2008, Issue 3. Art. No.: CD001539 |
| 52 | Smucny J; Becker LA; Glazier R. Beta2-agonists for acute bronchitis. Cochrane Database of Systematic Reviews. 2009, Issue 1. Art. No.: CD001726 |
| 53 | Singh M. Heated, humidified air for the common cold. Cochrane Database of Systematic Reviews. 2009, Issue 3. Art. No.: CD001728 |
| 54 | Medeiros IM; Saconato H. Antibiotic prophylaxis for mammalian bites. Cochrane Database of Systematic Reviews. 2008, Issue 3. Art. No.: CD001738 |
| 55 | Beamon SP; Falkenbach A; Linde K. Speleotherapy for asthma. Cochrane Database of Systematic Reviews. 2009, Issue 4. Art. No.: CD001741 |
| 56 | Tudur SC; Marson AG; Williamson PR. Phenytoin versus valproate monotherapy for partial onset seizures and generalized onset tonic-clonic seizures. Cochrane Database of Systematic Reviews. 2009, Issue 4. Art. No.: CD001769 |
| 57 | Lous J; Burton MJ; Felding J; Ovesen T; Rovers M; Williamson I. Grommets (ventilation tubes) for hearing loss associated with otitis media with effusion in children. Cochrane Database of Systematic Reviews. 2009, Issue 1. Art. No.: CD001801 |
| 58 | Burton MJ; Glasziou PP. Tonsillectomy or adeno-tonsillectomy versus non-surgical treatment for chronic/recurrent acute tonsillitis. Cochrane Database of Systematic Reviews. 2009, Issue 1. Art. No.: CD001802 |
| 59 | Smith SM; Schroeder K; Fahey T. Over-the-counter medications for acute cough in children and adults in ambulatory settings. Cochrane Database of Systematic Reviews. 2009, Issue 4. Art. No.: CD001831 |
| 60 | Prasad K; Kumar A; Singhal T; Gupta PK. Third generation cephalosporins versus conventional antibiotics for treating acute bacterial meningitis. Cochrane Database of Systematic Reviews. 2008, Issue 4. Art. No.: CD001832 |
| 61 | Macartney K; McIntyre P. Vaccines for post-exposure prophylaxis against varicella (chickenpox) in children and adults. Cochrane Database of Systematic Reviews. 2008, Issue 3. Art. No.: CD001833 |
| 62 | Conterno LO; da-Silva-Filho-Carlos-Rodrigues; Ruggeberg JU; Heath PT. Conjugate vaccines for preventing meningococcal C meningitis and septicaemia. Cochrane Database of Systematic Reviews. 2009, Issue 3. Art. No.: CD001834 |
| 63 | Thompson DC; Rivara F; Thompson R. Helmets for preventing head and facial injuries in bicyclists. Cochrane Database of Systematic Reviews. 2009, Issue 1. Art. No.: CD001855 |
| 64 | Mabey D; Fraser HN; Powell C. Antibiotics for trachoma. Cochrane Database of Systematic Reviews. 2009, Issue 1. Art. No.: CD001860 |
| 65 | Oude LH; Baur L; Jansen H; Shrewsbury VA; O'Malley C; Stolk RP; Summerbell CD. Interventions for treating obesity in children. Cochrane Database of Systematic Reviews. 2009, Issue 1. Art. No.: CD001872 |
| 66 | Br°nfort G; Nilsson N; Haas M; Evans RL; Goldsmith CH; ssendelft-Willem JJ; Bouter LM. Non-invasive physical treatments for chronic/recurrent headache. Cochrane Database of Systematic Reviews. 2009, Issue 1. Art. No.: CD001878 |
| 67 | Sirven J; Sperling MR; Wingerchuk DM. Early versus late antiepileptic drug withdrawal for people with epilepsy in remission. Cochrane Database of Systematic Reviews. 2010, Issue 4. Art. No.: CD001902 |
| 68 | Tudur SC; Marson AG; Williamson PR. Carbamazepine versus phenobarbitone monotherapy for epilepsy. Cochrane Database of Systematic Reviews. 2009, Issue 4. Art. No.: CD001904 |
| 69 | Appleton R; Macleod S; Martland T. Drug management for acute tonic-clonic convulsions including convulsive status epilepticus in children. Cochrane Database of Systematic Reviews. 2010, Issue 3. Art. No.: CD001905 |
| 70 | Tudur SC; Marson AG; Clough HE; Williamson PR. Carbamazepine versus phenytoin monotherapy for epilepsy. Cochrane Database of Systematic Reviews. 2010, Issue 3. Art. No.: CD001911 |
| 71 | Poustie VJ; Smyth RL; Watling RM. Oral protein calorie supplementation for children with chronic disease. Cochrane Database of Systematic Reviews. 2009, Issue 4. Art. No.: CD001914 |
| 72 | Vaile L; Williamson T; Waddell A; Taylor GJ. Interventions for ear discharge associated with grommets (ventilation tubes). Cochrane Database of Systematic Reviews. 2009, Issue 1. Art. No.: CD001933 |
| 73 | Thomas CL; Simpson S; Butler C; van d. Oral or topical nasal steroids for hearing loss associated with otitis media with effusion in children. Cochrane Database of Systematic Reviews. 2010, Issue 4. Art. No.: CD001935 |
| 74 | Panpanich R; Lerttrakarnnon P; Laopaiboon M. Azithromycin for acute lower respiratory tract infections. Cochrane Database of Systematic Reviews. 2008, Issue 4. Art. No.: CD001954 |
| 75 | Russell KF; Wiebe N; Saenz A; Ausejo SM; Johnson DW; Hartling L; Klassen TP. Glucocorticoids for croup. Cochrane Database of Systematic Reviews. 2009, Issue 1. Art. No.: CD001955 |
| 76 | Owen CM; Chalmers R; O'Sullivan T; Griffiths-Christopher EM. Antistreptococcal interventions for guttate and chronic plaque psoriasis. Cochrane Database of Systematic Reviews. 2009, Issue 1. Art. No.: CD001976 |
| 77 | Ramaratnam S; Baker GA; Goldstein LH. Psychological treatments for epilepsy. Cochrane Database of Systematic Reviews. 2009, Issue 1. Art. No.: CD002029 |
| 78 | Price KJ; Elliot TM. Stimulant laxatives for constipation and soiling in children. Cochrane Database of Systematic Reviews. 2008, Issue 4. Art. No.: CD002040 |
| 79 | Wasiak J; Cleland H; Campbell F. Dressings for superficial and partial thickness burns. Cochrane Database of Systematic Reviews. 2009, Issue 1. Art. No.: CD002106 |
| 80 | Bjerre LM; Verheij-Theo JM; Kochen MM. Antibiotics for community acquired pneumonia in adult outpatients. Cochrane Database of Systematic Reviews. 2009, Issue 4. Art. No.: CD002109 |
| 81 | Glazener-Cathryn MA; Evans-Jonathan HC. Desmopressin for nocturnal enuresis in children. Cochrane Database of Systematic Reviews. 2009, Issue 1. Art. No.: CD002112 |
| 82 | Glazener-Cathryn MA; Evans-Jonathan HC; Peto RE. Tricyclic and related drugs for nocturnal enuresis in children. Cochrane Database of Systematic Reviews. 2009, Issue 1. Art. No.: CD002117 |
| 83 | Meremikwu MM; Logan K; Garner P. Antipyretic measures for treating fever in malaria. Cochrane Database of Systematic Reviews. 2009, Issue 1. Art. No.: CD002151 |
| 84 | Campbell F; Gibson PG. Feather versus non-feather bedding for asthma. Cochrane Database of Systematic Reviews. 2009, Issue 3. Art. No.: CD002154 |
| 85 | Brocklebank-David DM; White J; Wright JJ; Jones P. Pressurised metered dose inhalers versus all other hand-held inhaler devices to deliver beta-2 agonist bronchodilators for non-acute asthma. Cochrane Database of Systematic Reviews. 2009, Issue 4. Art. No.: CD002158 |
| 86 | Sheikh A; Alves B; Dhami S. Pneumococcal vaccine for asthma. Cochrane Database of Systematic Reviews. 2010, Issue 1. Art. No.: CD002165 |
| 87 | Toelle B; Ram-Felix SF. Written individualised management plans for asthma in children and adults. Cochrane Database of Systematic Reviews. 2009, Issue 3. Art. No.: CD002171 |
| 88 | van-der-Wouden JC; Uijen-Johannes HJM; Bernsen R; Tasche-Marjolein JA; de-Jongste JC; Ducharme F. Inhaled sodium cromoglycate for asthma in children. Cochrane Database of Systematic Reviews. 2010, Issue 1. Art. No.: CD002173 |
| 89 | Rowe BH; Spooner C; Ducharme F; Bretzlaff J; Bota G. Early emergency department treatment of acute asthma with systemic corticosteroids. Cochrane Database of Systematic Reviews. 2008, Issue 4. Art. No.: CD002178 |
| 90 | Hayashi K; Yanagi M; Wood BR; Takamatsu I; nami-Keiko KA. Oxatomide for stable asthma in adults and children. Cochrane Database of Systematic Reviews. 2009, Issue 1. Art. No.: CD002179 |
| 91 | Taylor S; Tudur SC; Williamson PR; Marson AG. Phenobarbitone versus phenytoin monotherapy for partial onset seizures and generalized onset tonic-clonic seizures. Cochrane Database of Systematic Reviews. 2010, Issue 1. Art. No.: CD002217 |
| 92 | Manyemba J; Mayosi BM. Penicillin for secondary prevention of rheumatic fever. Cochrane Database of Systematic Reviews. 2009, Issue 4. Art. No.: CD002227 |
| 93 | Glazener-Cathryn MA; Evans-Jonathan HC; Peto RE. Drugs for nocturnal enuresis in children (other than desmopressin and tricyclics). Cochrane Database of Systematic Reviews. 2009, Issue 1. Art. No.: CD002238 |
| 94 | Brazzelli M; Griffiths P. Behavioural and cognitive interventions with or without other treatments for the management of faecal incontinence in children. Cochrane Database of Systematic Reviews. 2009, Issue 1. Art. No.: CD002240 |
| 95 | de BG; Hahn S; Borwick A. Antibiotic treatment for travellers' diarrhoea. Cochrane Database of Systematic Reviews. 2009, Issue 1. Art. No.: CD002242 |
| 96 | Marinho-Valeria CC; Higgins-Julian PT; Logan S; Sheiham A. Fluoride toothpastes for preventing dental caries in children and adolescents. Cochrane Database of Systematic Reviews. 2009, Issue 1. Art. No.: CD002278 |
| 97 | Robinson P; Deacon SA; Deery C; Heanue M; Walmsley AD; Worthington H; Glenny AM; Shaw BC. Manual versus powered toothbrushing for oral health. Cochrane Database of Systematic Reviews. 2009, Issue 1. Art. No.: CD002281 |
| 98 | Marinho-Valeria CC; Higgins-Julian PT; Logan S; Sheiham A. Fluoride mouthrinses for preventing dental caries in children and adolescents. Cochrane Database of Systematic Reviews. 2009, Issue 1. Art. No.: CD002284 |
| 99 | Augood C; MacLennan S; Gilbert RE; Logan S. Cisapride treatment for gastro-oesophageal reflux in children. Cochrane Database of Systematic Reviews. 2010, Issue 7. Art. No.: CD002300 |
| 100 | Spooner C; Spooner GR; Rowe BH. Mast-cell stabilising agents to prevent exercise-induced bronchoconstriction. Cochrane Database of Systematic Reviews. 2009, Issue 3. Art. No.: CD002307 |
| 101 | Edmonds M; Camargo CA; Pollack C; Rowe BH. Early use of inhaled corticosteroids in the emergency department treatment of acute asthma. Cochrane Database of Systematic Reviews. 2008, Issue 4. Art. No.: CD002308 |
| 102 | Adams NP; Lasserson TJ; Cates CJ; Jones P. Fluticasone versus beclomethasone or budesonide for chronic asthma in adults and children. Cochrane Database of Systematic Reviews. 2010, Issue 1. Art. No.: CD002310 |
| 103 | Ducharme F; di SF. Anti-leukotriene agents compared to inhaled corticosteroids in the management of recurrent and/or chronic asthma in adults and children. Cochrane Database of Systematic Reviews. 2008, Issue 4. Art. No.: CD002314 |
| 104 | Hazell P; O'Connell D; Heathcote D; Henry DA. Tricyclic drugs for depression in children and adolescents. Cochrane Database of Systematic Reviews. 2010, Issue 4. Art. No.: CD002317 |
| 105 | Kelly KD; Spooner C; Rowe BH. Nedocromil sodium versus sodium cromoglycate for preventing exercise-induced bronchoconstriction. Cochrane Database of Systematic Reviews. 2008, Issue 4. Art. No.: CD002731 |
| 106 | Adams NP; Bestall JC; Malouf R; Lasserson TJ; Jones P. Beclomethasone versus placebo for chronic asthma. Cochrane Database of Systematic Reviews. 2008, Issue 4. Art. No.: CD002738 |
| 107 | Koh MS; Tee A; Lasserson TJ; Irving LB. Inhaled corticosteroids compared to placebo for prevention of exercise induced bronchoconstriction. Cochrane Database of Systematic Reviews. 2009, Issue 2. Art. No.: CD002739 |
| 108 | Graham V; Lasserson TJ; Rowe BH. Antibiotics for acute asthma. Cochrane Database of Systematic Reviews. 2009, Issue 1. Art. No.: CD002741 |
| 109 | Matheson NJ; Harnden A; Perera R; Sheikh A; Symmonds AM. Neuraminidase inhibitors for preventing and treating influenza in children. Cochrane Database of Systematic Reviews. 2008, Issue 4. Art. No.: CD002744 |
| 110 | ves-Galvπo MG; Rocha-Crispino-Santos-Marilene-Augusta; Alves-da-Cunha-Antonio-JL. Amantadine and rimantadine for influenza A in children and the elderly. Cochrane Database of Systematic Reviews. 2009, Issue 3. Art. No.: CD002745 |
| 111 | Victor S; Ryan S. Drugs for preventing migraine headaches in children. Cochrane Database of Systematic Reviews. 2008, Issue 3. Art. No.: CD002761 |
| 112 | Marinho-Valeria CC; Higgins-Julian PT; Sheiham A; Logan S. One topical fluoride (toothpastes, or mouthrinses, or gels, or varnishes) versus another for preventing dental caries in children and adolescents. Cochrane Database of Systematic Reviews. 2009, Issue 1. Art. No.: CD002780 |
| 113 | Marinho-Valeria CC; Higgins-Julian PT; Sheiham A; Logan S. Combinations of topical fluoride (toothpastes, mouthrinses, gels, varnishes) versus single topical fluoride for preventing dental caries in children and adolescents. Cochrane Database of Systematic Reviews. 2009, Issue 1. Art. No.: CD002781 |
| 114 | Marinho-Valeria CC; Higgins-Julian PT; Logan S; Sheiham A. Topical fluoride (toothpastes, mouthrinses, gels or varnishes) for preventing dental caries in children and adolescents. Cochrane Database of Systematic Reviews. 2009, Issue 1. Art. No.: CD002782 |
| 115 | Hahn S; Kim Y; Garner P. Reduced osmolarity oral rehydration solution for treating dehydration caused by acute diarrhoea in children. Cochrane Database of Systematic Reviews. 2009, Issue 1. Art. No.: CD002847 |
| 116 | Soares WK; Goldberg E; Tamimi G; Leibovici L; Pitan F. Rotavirus vaccine for preventing diarrhoea. Cochrane Database of Systematic Reviews. 2009, Issue 4. Art. No.: CD002848 |
| 117 | Spinks A; Wasiak J; Villanueva E; Bernath V. Scopolamine (hyoscine) for preventing and treating motion sickness. Cochrane Database of Systematic Reviews. 2009, Issue 4. Art. No.: CD002851 |
| 118 | Moore M; Little P. Humidified air inhalation for treating croup. Cochrane Database of Systematic Reviews. 2008, Issue 4. Art. No.: CD002870 |
| 119 | Chavasse-Richard JPG; Seddon P; Bara A; McKean MC. Short acting beta2-agonists for recurrent wheeze in children under two years of age. Cochrane Database of Systematic Reviews. 2009, Issue 1. Art. No.: CD002873 |
| 120 | Shneerson J; Wright JJ. Lifestyle modification for obstructive sleep apnoea. Cochrane Database of Systematic Reviews. 2009, Issue 4. Art. No.: CD002875 |
| 121 | Adams NP; Bestall JC; Jones P. Beclomethasone at different doses for chronic asthma. Cochrane Database of Systematic Reviews. 2009, Issue 3. Art. No.: CD002879 |
| 122 | Seddon P; Bara A; Lasserson TJ; Ducharme FM. Oral xanthines as maintenance treatment for asthma in children. Cochrane Database of Systematic Reviews. 2009, Issue 1. Art. No.: CD002885 |
| 123 | Wilson D; Torres LM; Durham S. Sublingual immunotherapy for allergic rhinitis. Cochrane Database of Systematic Reviews. 2009, Issue 1. Art. No.: CD002893 |
| 124 | Glazener-Cathryn MA; Evans-Jonathan HC; Peto RE. Alarm interventions for nocturnal enuresis in children. Cochrane Database of Systematic Reviews. 2009, Issue 1. Art. No.: CD002911 |
| 125 | Coren E; Barlow J. Individual and group-based parenting programmes for improving psychosocial outcomes for teenage parents and their children. Cochrane Database of Systematic Reviews. 2009, Issue 1. Art. No.: CD002964 |
| 126 | Klassen TP; Hartling L. Acyclovir for treating varicella in otherwise healthy children and adolescents. Cochrane Database of Systematic Reviews. 2010, Issue 7. Art. No.: CD002980 |
| 127 | Blackhall K; Appleton S; Cates CJ. Ionisers for chronic asthma. Cochrane Database of Systematic Reviews. 2010, Issue 2. Art. No.: CD002986 |
| 128 | Kilburn SA; Lasserson TJ; McKean MC. Pet allergen control measures for allergic asthma in children and adults. Cochrane Database of Systematic Reviews. 2009, Issue 1. Art. No.: CD002989 |
| 129 | Richeldi L; Ferrara G; Fabbri L; Lasserson TJ; Gibson PG. Macrolides for chronic asthma. Cochrane Database of Systematic Reviews. 2008, Issue 4. Art. No.: CD002997 |
| 130 | Huertas-Ceballos AA; Logan S; Bennett C; Macarthur C. Psychosocial interventions for recurrent abdominal pain (RAP) and irritable bowel syndrome (IBS) in childhood. Cochrane Database of Systematic Reviews. 2009, Issue 1. Art. No.: CD003014 |
| 131 | Huertas-Ceballos AA; Logan S; Bennett C; Macarthur C. Pharmacological interventions for recurrent abdominal pain (RAP) and irritable bowel syndrome (IBS) in childhood. Cochrane Database of Systematic Reviews. 2009, Issue 1. Art. No.: CD003017 |
| 132 | Huertas-Ceballos AA; Logan S; Bennett C; Macarthur C. Dietary interventions for recurrent abdominal pain (RAP) and irritable bowel syndrome (IBS) in childhood. Cochrane Database of Systematic Reviews. 2009, Issue 1. Art. No.: CD003019 |
| 133 | Posner EB; Mohamed KK; Marson AG. Ethosuximide, sodium valproate or lamotrigine for absence seizures in children and adolescents. Cochrane Database of Systematic Reviews. 2010, Issue 3. Art. No.: CD003032 |
| 134 | Allen SJ; Okoko B; Martinez EG; Gregorio G; Dans LF. Probiotics for treating infectious diarrhoea. Cochrane Database of Systematic Reviews. 2009, Issue 1. Art. No.: CD003048 |
| 135 | Orozco LJ; Buchleitner AM; Gimenez PG; Figuls M; Richter B; Mauricio D. Exercise or exercise and diet for preventing type 2 diabetes mellitus. Cochrane Database of Systematic Reviews. 2008, Issue 3. Art. No.: CD003054 |
| 136 | Hartling L; Wiebe N; Russell KF; Patel H; Klassen TP. Epinephrine for bronchiolitis. Cochrane Database of Systematic Reviews. 2009, Issue 4. Art. No.: CD003123 |
| 137 | Duijvestijn-Yvonne CM; Mourdi N; Smucny J; Pons G; Chalumeau M. Acetylcysteine and carbocysteine for acute upper and lower respiratory tract infections in paediatric patients without chronic broncho-pulmonary disease. Cochrane Database of Systematic Reviews. 2009, Issue 1. Art. No.: CD003124 |
| 138 | Ducharme F. Addition of anti-leukotriene agents to inhaled corticosteroids for chronic asthma. Cochrane Database of Systematic Reviews. 2010, Issue 1. Art. No.: CD003133 |
| 139 | Adams NP; Bestall JC; Lasserson TJ; Jones P; Cates CJ. Fluticasone versus placebo for chronic asthma in adults and children. Cochrane Database of Systematic Reviews. 2009, Issue 4. Art. No.: CD003135 |
| 140 | Ducharme F; Lasserson TJ; Cates CJ. Long-acting beta2-agonists versus anti-leukotrienes as add-on therapy to inhaled corticosteroids for chronic asthma. Cochrane Database of Systematic Reviews. 2009, Issue 3. Art. No.: CD003137 |
| 141 | Al-Sayyad JJ; Fedorowicz Z; Alhashimi D; Jamal A. Topical nasal steroids for intermittent and persistent allergic rhinitis in children. Cochrane Database of Systematic Reviews. 2009, Issue 1. Art. No.: CD003163 |
| 142 | Wu T; Liu GJ; Li P; Clar C. Iodised salt for preventing iodine deficiency disorders. Cochrane Database of Systematic Reviews. 2009, Issue 1. Art. No.: CD003204 |
| 143 | Pohl A. Modes of administration of antibiotics for symptomatic severe urinary tract infections. Cochrane Database of Systematic Reviews. 2008, Issue 4. Art. No.: CD003237 |
| 144 | Koning S; Verhagen AP; van-Suijlekom-Smit-Lisette-WA; Morris AD; Butler C; van-der-Wouden JC. Interventions for impetigo. Cochrane Database of Systematic Reviews. 2009, Issue 1. Art. No.: CD003261 |
| 145 | Adams NP; Bestall JC; Jones P. Budesonide at different doses for chronic asthma. Cochrane Database of Systematic Reviews. 2008, Issue 4. Art. No.: CD003271 |
| 146 | Yorke J; Fleming SL; Shuldham C. Psychological interventions for children with asthma. Cochrane Database of Systematic Reviews. 2009, Issue 3. Art. No.: CD003272 |
| 147 | Adams NP; Bestall JC; Jones P. Budesonide versus placebo for chronic asthma in children and adults. Cochrane Database of Systematic Reviews. 2008, Issue 4. Art. No.: CD003274 |
| 148 | Grimshaw G; Stanton A. Tobacco cessation interventions for young people. Cochrane Database of Systematic Reviews. 2010, Issue 1. Art. No.: CD003289 |
| 149 | Jones L;Watling RM;Wilkins S;Pizer B;. Nutritional support in children and young people with cancer undergoing chemotherapy. Cochrane Database of Systematic Reviews. 2010, Issue 7. Art. No.: CD003298 |
| 150 | Farion KJ; Russell KF; Osmond MH; Hartling L; Klassen TP; Durec T; Vandermeer B. Tissue adhesives for traumatic lacerations in children and adults. Cochrane Database of Systematic Reviews. 2009, Issue 1. Art. No.: CD003326 |
| 151 | Merry SN; McDowell HH; Hetrick SE; Bir JJ; Muller N. Psychological and/or educational interventions for the prevention of depression in children and adolescents. Cochrane Database of Systematic Reviews. 2009, Issue 1. Art. No.: CD003380 |
| 152 | Hassiotis AA; Hall I. Behavioural and cognitive-behavioural interventions for outwardly-directed aggressive behaviour in people with learning disabilities. Cochrane Database of Systematic Reviews. 2009, Issue 3. Art. No.: CD003406 |
| 153 | Griffin G; Flynn CA; Bailey RE; Schultz JK. Antihistamines and/or decongestants for otitis media with effusion (OME) in children. Cochrane Database of Systematic Reviews. 2009, Issue 1. Art. No.: CD003423 |
| 154 | Nelson RL. Non surgical therapy for anal fissure. Cochrane Database of Systematic Reviews. 2008, Issue 4. Art. No.: CD003431 |
| 155 | Quigley C. Hydromorphone for acute and chronic pain. Cochrane Database of Systematic Reviews. 2009, Issue 1. Art. No.: CD003447 |
| 156 | Villar JC; Villar LA; Marin-Neto JA; Ebrahim S; Yusuf S. Trypanocidal drugs for chronic asymptomatic Trypanosoma cruzi infection. Cochrane Database of Systematic Reviews. 2009, Issue 1. Art. No.: CD003463 |
| 157 | Pennington L; Goldbart J; Marshall J. Speech and language therapy to improve the communication skills of children with cerebral palsy. Cochrane Database of Systematic Reviews. 2009, Issue 1. Art. No.: CD003466 |
| 158 | Heintjes EM; Berger M; Bierma-Zeinstra-Sita MA; Bernsen R; Verhaar-Jan AN; Koes BW. Pharmacotherapy for patellofemoral pain syndrome. Cochrane Database of Systematic Reviews. 2008, Issue 4. Art. No.: CD003470 |
| 159 | Heintjes EM; Berger M; Bierma-Zeinstra-Sita MA; Bernsen R; Verhaar-Jan AN; Koes BW. Exercise therapy for patellofemoral pain syndrome. Cochrane Database of Systematic Reviews. 2009, Issue 1. Art. No.: CD003472 |
| 160 | Oniyangi O; Omari-Aika AA. Malaria chemoprophylaxis in sickle cell disease. Cochrane Database of Systematic Reviews. 2009, Issue 1. Art. No.: CD003489 |
| 161 | Nye C; Brice A. Combined vitamin B6-magnesium treatment in autism spectrum disorder. Cochrane Database of Systematic Reviews. 2009, Issue 1. Art. No.: CD003497 |
| 162 | Millward C; Ferriter M; Calver SJ; Connell-Jones GG. Gluten- and casein-free diets for autistic spectrum disorder. Cochrane Database of Systematic Reviews. 2009, Issue 1. Art. No.: CD003498 |
| 163 | Grimwade K; Swingler GH. Cotrimoxazole prophylaxis for opportunistic infections in children with HIV infection. Cochrane Database of Systematic Reviews. 2009, Issue 1. Art. No.: CD003508 |
| 164 | Adams NP; Bestall JC; Jones P. Beclomethasone versus budesonide for chronic asthma. Cochrane Database of Systematic Reviews. 2008, Issue 4. Art. No.: CD003530 |
| 165 | Adams NP; Bestall JC; Jones P; Lasserson TJ; Griffiths B; Cates CJ. Fluticasone at different doses for chronic asthma in adults and children. Cochrane Database of Systematic Reviews. 2009, Issue 3. Art. No.: CD003534 |
| 166 | McDonald N; Bara A; McKean MC. Anticholinergic therapy for chronic asthma in children over two years of age. Cochrane Database of Systematic Reviews. 2010, Issue 3. Art. No.: CD003535 |
| 167 | Allam MF; Lucena RA. Selenium supplementation for asthma. Cochrane Database of Systematic Reviews. 2009, Issue 1. Art. No.: CD003538 |
| 168 | Guevara JP; Ducharme F; Keren R; Nihtianova S; Zorc J. Inhaled corticosteroids versus sodium cromoglycate in children and adults with asthma. Cochrane Database of Systematic Reviews. 2009, Issue 3. Art. No.: CD003558 |
| 169 | Singh M; Bara A; Gibson PG. Humidity control for chronic asthma. Cochrane Database of Systematic Reviews. 2008, Issue 4. Art. No.: CD003563 |
| 170 | Taramarcaz P; Gibson PG. Intranasal corticosteroids for asthma control in people with coexisting asthma and rhinitis. Cochrane Database of Systematic Reviews. 2009, Issue 3. Art. No.: CD003570 |
| 171 | Cardwell ME; Siviter G; Smith AF. Non-steroidal anti-inflammatory drugs and perioperative bleeding in paediatric tonsillectomy. Cochrane Database of Systematic Reviews. 2008, Issue 4. Art. No.: CD003591 |
| 172 | Kapczinski-Flavio FK; Silva-de LM; dos-Santos-Souza-Juliano-JSS; Batista-Miralha-da-Cunha-Angelo-ABC; Schmitt-Ricardo RS. Antidepressants for generalized anxiety disorder. Cochrane Database of Systematic Reviews. 2009, Issue 1. Art. No.: CD003592 |
| 173 | Muller M; Marson AG; Williamson PR. Oxcarbazepine versus phenytoin monotherapy for epilepsy. Cochrane Database of Systematic Reviews. 2009, Issue 4. Art. No.: CD003615 |
| 174 | Glazener-Cathryn MA; Evans-Jonathan HC. Simple behavioural and physical interventions for nocturnal enuresis in children. Cochrane Database of Systematic Reviews. 2009, Issue 1. Art. No.: CD003637 |
| 175 | Irlam-James JH; Visser-Marianne ME; Rollins NN; Siegfried N. Micronutrient supplementation in children and adults with HIV infection. Cochrane Database of Systematic Reviews. 2009, Issue 1. Art. No.: CD003650 |
| 176 | Ejere-Henry OD; Alhassan MB; Rabiu M. Face washing promotion for preventing active trachoma. Cochrane Database of Systematic Reviews. 2009, Issue 1. Art. No.: CD003659 |
| 177 | Meremikwu MM; Oyo IA. Paracetamol versus placebo or physical methods for treating fever in children. Cochrane Database of Systematic Reviews. 2009, Issue 2. Art. No.: CD003676 |
| 178 | Ekeland E; Heian F; Hagen KB; Abbott JM; Nordheim L. Exercise to improve self-esteem in children and young people. Cochrane Database of Systematic Reviews. 2009, Issue 1. Art. No.: CD003683 |
| 179 | Hatt SR; Gnanaraj L. Interventions for intermittent exotropia. Cochrane Database of Systematic Reviews. 2009, Issue 2. Art. No.: CD003737 |
| 180 | Addiss D; Gamble CL; Garner P; Gelband H; Ejere-Henry OD; Critchley JA; International-Filariasis-Review-Group. Albendazole for lymphatic filariasis. Cochrane Database of Systematic Reviews. 2009, Issue 1. Art. No.: CD003753 |
| 181 | Murphy CK; Hahn S; Volmink J. Reduced osmolarity oral rehydration solution for treating cholera. Cochrane Database of Systematic Reviews. 2009, Issue 1. Art. No.: CD003754 |
| 182 | Meremikwu MM; Donegan S; Esu E. Chemoprophylaxis and intermittent treatment for preventing malaria in children. Cochrane Database of Systematic Reviews. 2009, Issue 1. Art. No.: CD003756 |
| 183 | Hodson EM; Willis NS; Craig JC. Antibiotics for acute pyelonephritis in children. Cochrane Database of Systematic Reviews. 2010, Issue 3. Art. No.: CD003772 |
| 184 | Angermayr L; Clar C. Iodine supplementation for preventing iodine deficiency disorders in children. Cochrane Database of Systematic Reviews. 2009, Issue 1. Art. No.: CD003819 |
| 185 | Fernandez R; Griffiths R. Water for wound cleansing. Cochrane Database of Systematic Reviews. 2010, Issue 5. Art. No.: CD003861 |
| 186 | Birnie AJ; Bath-Hextall FJ; Ravenscroft JC; Williams HC. Interventions to reduce Staphylococcus aureus in the management of atopic eczema. Cochrane Database of Systematic Reviews. 2008, Issue 3. Art. No.: CD003871 |
| 187 | Blitz M; Blitz S; Beasely R; Diner B; Hughes R; Knopp JA; Rowe BH. Inhaled magnesium sulfate in the treatment of acute asthma. Cochrane Database of Systematic Reviews. 2009, Issue 3. Art. No.: CD003898 |
| 188 | Walters EH; Walters-Julia AE; Gibson PG. Regular treatment with long acting beta agonists versus daily regular treatment with short acting beta agonists in adults and children with stable asthma. Cochrane Database of Systematic Reviews. 2009, Issue 1. Art. No.: CD003901 |
| 189 | Liu JP; Manheimer E; Yang M. Herbal medicines for treating HIV infection and AIDS. Cochrane Database of Systematic Reviews. 2009, Issue 1. Art. No.: CD003937 |
| 190 | Pienaar ED; Young T; Holmes H. Interventions for the prevention and management of oropharyngeal candidiasis associated with HIV infection in adults and children. Cochrane Database of Systematic Reviews. 2009, Issue 1. Art. No.: CD003940 |
| 191 | Jacobson-Vann JC; Szilagyi P. Patient reminder and recall systems to improve immunization rates. Cochrane Database of Systematic Reviews. 2009, Issue 1. Art. No.: CD003941 |
| 192 | Evans BW; Clark WK; Moore DJ; Whorwell PJ. Tegaserod for the treatment of irritable bowel syndrome and chronic constipation. Cochrane Database of Systematic Reviews. 2008, Issue 4. Art. No.: CD003960 |
| 193 | Eccleston C; Palermo TM; Williams-Amanda Cd; Lewandowski A; Morley S. Psychological therapies for the management of chronic and recurrent pain in children and adolescents. Cochrane Database of Systematic Reviews. 2009, Issue 2. Art. No.: CD003968 |
| 194 | Macritchie K; Geddes J; Scott J; Haslam DR; Silva-de LM; Goodwin G. Valproate for acute mood episodes in bipolar disorder. Cochrane Database of Systematic Reviews. 2009, Issue 1. Art. No.: CD004052 |
| 195 | Ersser SJ; Latter S; Sibley A; Satherley PA; Welbourne S. Psychological and educational interventions for atopic eczema in children. Cochrane Database of Systematic Reviews. 2009, Issue 1. Art. No.: CD004054 |
| 196 | Clar C; Waugh N; Thomas S. Routine hospital admission versus out-patient or home care in children at diagnosis of type 1 diabetes mellitus. Cochrane Database of Systematic Reviews. 2009, Issue 1. Art. No.: CD004099 |
| 197 | Lasserson TJ; Cates CJ; Ferrara G; Casali L. Combination fluticasone and salmeterol versus fixed dose combination budesonide and formoterol for chronic asthma in adults and children. Cochrane Database of Systematic Reviews. 2010, Issue 3. Art. No.: CD004106 |
| 198 | Sridhar A; McKean MC. Nedocromil sodium for chronic asthma in children. Cochrane Database of Systematic Reviews. 2010, Issue 1. Art. No.: CD004108 |
| 199 | Powell H; Gibson PG. High dose versus low dose inhaled corticosteroid as initial starting dose for asthma in adults and children. Cochrane Database of Systematic Reviews. 2008, Issue 4. Art. No.: CD004109 |
| 200 | Law J; Garrett Z; Nye C. Speech and language therapy interventions for children with primary speech and language delay or disorder. Cochrane Database of Systematic Reviews. 2010, Issue 5. Art. No.: CD004110 |
| 201 | Hoare BJ; Wasiak J; Imms C; Carey L. Constraint-induced movement therapy in the treatment of the upper limb in children with hemiplegic cerebral palsy. Cochrane Database of Systematic Reviews. 2009, Issue 1. Art. No.: CD004149 |
| 202 | Simpson S; Thomas CL; van-der LM; MacMillan H; van-der-Wouden JC; Butler C. Identification of children in the first four years of life for early treatment for otitis media with effusion. Cochrane Database of Systematic Reviews. 2010, Issue 1. Art. No.: CD004163 |
| 203 | Liu JP; Nikolova D; Fei Y. Immunoglobulins for preventing hepatitis A. Cochrane Database of Systematic Reviews. 2009, Issue 2. Art. No.: CD004181 |
| 204 | Tomerak-Ahmed AT; McGlashan J; Lakhanpaul M; Vyas-Harish HV; McKean MC. Inhaled corticosteroids for non-specific chronic cough in children. Cochrane Database of Systematic Reviews. 2009, Issue 3. Art. No.: CD004231 |
| 205 | Lander JA; Weltman BJ; So SS. EMLA and Amethocaine for reduction of children's pain associated with needle insertion. Cochrane Database of Systematic Reviews. 2009, Issue 1. Art. No.: CD004236 |
| 206 | Baer EL; Davies MW; Easterbrook K. Disposable nappies for preventing napkin dermatitis in infants. Cochrane Database of Systematic Reviews. 2009, Issue 3. Art. No.: CD004262 |
| 207 | Meremikwu MM; Oyo IA. Physical methods versus drug placebo or no treatment for managing fever in children. Cochrane Database of Systematic Reviews. 2009, Issue 2. Art. No.: CD004264 |
| 208 | Kilburn SA;Featherstone P;Higgins B;Brindle R;. Interventions for cellulitis and erysipelas. Cochrane Database of Systematic Reviews. 2010, Issue 6. Art. No.: CD004299 |
| 209 | Davies MW; Dore AJ; Perissinotto KL. Topical Vitamin A, or its derivatives, for treating and preventing napkin dermatitis in infants. Cochrane Database of Systematic Reviews. 2009, Issue 3. Art. No.: CD004300 |
| 210 | Burton MJ; Doree C. Ear drops for the removal of ear wax. Cochrane Database of Systematic Reviews. 2009, Issue 1. Art. No.: CD004326 |
| 211 | Chang AB; McKean MC; Morris PS. Anticholinergics for prolonged non-specific cough in children. Cochrane Database of Systematic Reviews. 2010, Issue 5. Art. No.: CD004358 |
| 212 | Lane DA; Millane TA; Lip-Gregory YH. Psychological interventions for depression in adolescent and adult congenital heart disease. Cochrane Database of Systematic Reviews. 2009, Issue 1. Art. No.: CD004372 |
| 213 | Gold C; Wigram T; Elefant C. Music therapy for autistic spectrum disorder. Cochrane Database of Systematic Reviews. 2010, Issue 1. Art. No.: CD004381 |
| 214 | Bukirwa H; Garner P; Critchley JA. Chlorproguanil-dapsone for treating uncomplicated malaria. Cochrane Database of Systematic Reviews. 2010, Issue 1. Art. No.: CD004387 |
| 215 | Galappaththy-Gawrie NL; Omari-Aika AA; Tharyan P. Primaquine for preventing relapses in people with Plasmodium vivax malaria. Cochrane Database of Systematic Reviews. 2008, Issue 3. Art. No.: CD004389 |
| 216 | Hartling L; Bellemare S; Wiebe N; Russell KF; Klassen TP; Craig WR. Oral versus intravenous rehydration for treating dehydration due to gastroenteritis in children. Cochrane Database of Systematic Reviews. 2010, Issue 1. Art. No.: CD004390 |
| 217 | Leach AJ; Morris PS. Antibiotics for the prevention of acute and chronic suppurative otitis media in children. Cochrane Database of Systematic Reviews. 2009, Issue 4. Art. No.: CD004401 |
| 218 | Candy B; Hotopf M. Steroids for symptom control in infectious mononucleosis. Cochrane Database of Systematic Reviews. 2010, Issue 7. Art. No.: CD004402 |
| 219 | Altunaiji SM; Kukuruzovic RH; Curtis NC; Massie J. Antibiotics for whooping cough (pertussis). Cochrane Database of Systematic Reviews. 2009, Issue 3. Art. No.: CD004404 |
| 220 | van-de BD; de GJ; McIntyre P; Prasad K. Corticosteroids for acute bacterial meningitis. Cochrane Database of Systematic Reviews. 2008, Issue 4. Art. No.: CD004405 |
| 221 | Demicheli V; Jefferson T; Rivetti A; Price D. Vaccines for measles, mumps and rubella in children. Cochrane Database of Systematic Reviews. 2008, Issue 4. Art. No.: CD004407 |
| 222 | Spurling-Geoffrey KP; Del MC; Dooley L; Foxlee R. Delayed antibiotics for respiratory infections. Cochrane Database of Systematic Reviews. 2010, Issue 1. Art. No.: CD004417 |
| 223 | Guppy-Michelle PB; Mickan SM; Del MC. Advising patients to increase fluid intake for treating acute respiratory infections. Cochrane Database of Systematic Reviews. 2010, Issue 2. Art. No.: CD004419 |
| 224 | Chang AB; Marchant JM; McKean MC; Morris PS. Inhaled cromones for prolonged non-specific cough in children. Cochrane Database of Systematic Reviews. 2009, Issue 4. Art. No.: CD004436 |
| 225 | Burton MJ; Doree C. Interventions for recurrent idiopathic epistaxis (nosebleeds) in children. Cochrane Database of Systematic Reviews. 2009, Issue 1. Art. No.: CD004461 |
| 226 | Osei AA; Orton LC; Owusu OS. Atovaquone-proguanil for treating uncomplicated malaria. Cochrane Database of Systematic Reviews. 2009, Issue 1. Art. No.: CD004529 |
| 227 | Thaver D; Zaidi-Anita KM; Critchley JA; Azmatullah A; Madni SA; Bhutta ZA. Fluoroquinolones for treating typhoid and paratyphoid fever (enteric fever). Cochrane Database of Systematic Reviews. 2008, Issue 4. Art. No.: CD004530 |
| 228 | Bukirwa H; Orton LC. Artesunate plus mefloquine versus mefloquine for treating uncomplicated malaria. Cochrane Database of Systematic Reviews. 2010, Issue 1. Art. No.: CD004531 |
| 229 | Chen X; Wu T; Liu G; Wang Q; Zheng J; Wei J; Ni J; Zhou L; Duan X; Qiao J. Chinese medicinal herbs for influenza. Cochrane Database of Systematic Reviews. 2010, Issue 2. Art. No.: CD004559 |
| 230 | Handoll-Helen HG; Vaghela M. Interventions for treating mallet finger injuries. Cochrane Database of Systematic Reviews. 2008, Issue 3. Art. No.: CD004574 |
| 231 | Mason DL; Dickens VA; Vail A. Rehabilitation for hamstring injuries. Cochrane Database of Systematic Reviews. 2008, Issue 4. Art. No.: CD004575 |
| 232 | Macfadyen CA; Acuin JM; Gamble CL. Topical antibiotics without steroids for chronically discharging ears with underlying eardrum perforations. Cochrane Database of Systematic Reviews. 2009, Issue 1. Art. No.: CD004618 |
| 233 | Glazener-Cathryn MA; Evans-Jonathan HC; Peto RE. Complex behavioural and educational interventions for nocturnal enuresis in children. Cochrane Database of Systematic Reviews. 2008, Issue 3. Art. No.: CD004668 |
| 234 | Cheng J; Pan T. Calorie controlled diet for chronic asthma. Cochrane Database of Systematic Reviews. 2008, Issue 4. Art. No.: CD004674 |
| 235 | Williams K;Wheeler DM;Silove N;Hazell P;. Selective serotonin reuptake inhibitors (SSRIs) for autism spectrum disorders (ASD). Cochrane Database of Systematic Reviews. 2010, Issue 8. Art. No.: CD004677 |
| 236 | Gonzßlez U; Seaton T; Bergus G; Jacobson J; Martφnez MC. Systemic antifungal therapy for tinea capitis in children. Cochrane Database of Systematic Reviews. 2009, Issue 3. Art. No.: CD004685 |
| 237 | James-Anthony ACJ; Soler A; Weatherall-Rosamund RW. Cognitive behavioural therapy for anxiety disorders in children and adolescents. Cochrane Database of Systematic Reviews. 2009, Issue 1. Art. No.: CD004690 |
| 238 | Larun L; Nordheim L; Ekeland E; Hagen KB; Heian F. Exercise in prevention and treatment of anxiety and depression among children and young people. Cochrane Database of Systematic Reviews. 2009, Issue 1. Art. No.: CD004691 |
| 239 | McDonald S; Langton-Hewer CD; Nunez DA. Grommets (ventilation tubes) for recurrent acute otitis media in children. Cochrane Database of Systematic Reviews. 2008, Issue 4. Art. No.: CD004741 |
| 240 | van-der-Wouden JC; Menke J; Gajadin S; Koning S; Tasche-Marjolein JA; van-Suijlekom-Smit-Lisette-WA; Berger M; Butler C. Interventions for cutaneous molluscum contagiosum. Cochrane Database of Systematic Reviews. 2010, Issue 2. Art. No.: CD004767 |
| 241 | Fisher CA;Hetrick SE;Rushford N;. Family therapy for anorexia nervosa. Cochrane Database of Systematic Reviews. 2010, Issue 6. Art. No.: CD004780 |
| 242 | Zhang X; Wu T; Zhang J; Yan Q; Xie L; Liu GJ. Chinese medicinal herbs for the common cold. Cochrane Database of Systematic Reviews. 2010, Issue 7. Art. No.: CD004782 |
| 243 | Marchant JM; Morris PS; Gaffney J; Chang AB. Antibiotics for prolonged moist cough in children. Cochrane Database of Systematic Reviews. 2008, Issue 3. Art. No.: CD004822 |
| 244 | Chang AB; Lasserson TJ; Gaffney J; Connor FL; Garske LA. Gastro-oesophageal reflux treatment for prolonged non-specific cough in children and adults. Cochrane Database of Systematic Reviews. 2009, Issue 4. Art. No.: CD004823 |
| 245 | Johnston BC; Supina AL; Ospina M; Vohra S. Probiotics for the prevention of pediatric antibiotic-associated diarrhea. Cochrane Database of Systematic Reviews. 2008, Issue 4. Art. No.: CD004827 |
| 246 | Ramacciotti AS; Soares B; Atallah ┴N. Dipyrone for acute primary headaches. Cochrane Database of Systematic Reviews. 2009, Issue 1. Art. No.: CD004842 |
| 247 | Hetrick SE; Merry SN; McKenzie J; Sindahl P; Proctor M. Selective serotonin reuptake inhibitors (SSRIs) for depressive disorders in children and adolescents. Cochrane Database of Systematic Reviews. 2009, Issue 1. Art. No.: CD004851 |
| 248 | O'Kearney RT; Anstey K; von SC. Behavioural and cognitive behavioural therapy for obsessive compulsive disorder in children and adolescents. Cochrane Database of Systematic Reviews. 2010, Issue 1. Art. No.: CD004856 |
| 249 | Altamimi S; Khalil A; Khalaiwi KA; Milner R; Pusic M; Al-Othman MA. Short versus standard duration antibiotic therapy for acute streptococcal pharyngitis in children. Cochrane Database of Systematic Reviews. 2010, Issue 7. Art. No.: CD004872 |
| 250 | Kabra SK; Lodha R; Pandey RM. Antibiotics for community acquired pneumonia in children. Cochrane Database of Systematic Reviews. 2010, Issue 3. Art. No.: CD004874 |
| 251 | Gavranich JB; Chang AB. Antibiotics for community acquired lower respiratory tract infections (LRTI) secondary to Mycoplasma pneumoniae in children. Cochrane Database of Systematic Reviews. 2010, Issue 7. Art. No.: CD004875 |
| 252 | Jefferson T; Rivetti A; Harnden A; Di PC; Demicheli V. Vaccines for preventing influenza in healthy children. Cochrane Database of Systematic Reviews. 2008, Issue 2. Art. No.: CD004879 |
| 253 | Blom-Danielle JM; Ermers M; Bont L; van-Woensel-Job BM; Van-Aalderen-Wim MC. Inhaled corticosteroids during acute bronchiolitis in the prevention of post-bronchiolitic wheezing. Cochrane Database of Systematic Reviews. 2009, Issue 3. Art. No.: CD004881 |
| 254 | Elliott S; Shafiq A. Interventions for infantile esotropia. Cochrane Database of Systematic Reviews. 2009, Issue 1. Art. No.: CD004917 |
| 255 | Bukirwa H; Critchley JA. Sulfadoxine-pyrimethamine plus artesunate versus sulfadoxine-pyrimethamine plus amodiaquine for treating uncomplicated malaria. Cochrane Database of Systematic Reviews. 2009, Issue 1. Art. No.: CD004966 |
| 256 | -Del-Rio-Navarro-Blanca-Estela; Espinosa-Rosales FJ; Flenady V; Sienra-Monge-Juan JL. Immunostimulants for preventing respiratory tract infection in children. Cochrane Database of Systematic Reviews. 2010, Issue 3. Art. No.: CD004974 |
| 257 | Thanaviratananich S; Laopaiboon M; Vatanasapt P. Once or twice daily versus three times daily amoxicillin with or without clavulanate for the treatment of acute otitis media. Cochrane Database of Systematic Reviews. 2010, Issue 2. Art. No.: CD004975 |
| 258 | Lucero MG;Dulalia VE;Nillos LT;Williams G;Parre±o-Rhea AN;Nohynek H;Riley ID;Makela H;. Pneumococcal conjugate vaccines for preventing vaccine-type invasive pneumococcal disease and X-ray defined pneumonia in children less than two years of age. Cochrane Database of Systematic Reviews. 2009, Issue 4. Art. No.: CD004977 |
| 259 | Ranganathan LN; Ramaratnam S. Rapid versus slow withdrawal of antiepileptic drugs. Cochrane Database of Systematic Reviews. 2009, Issue 4. Art. No.: CD005003 |
| 260 | Powell C; Hatt SR. Vision screening for amblyopia in childhood. Cochrane Database of Systematic Reviews. 2009, Issue 3. Art. No.: CD005020 |
| 261 | Mason AR; Mason J; Cork M; Dooley G; Edwards G. Topical treatments for chronic plaque psoriasis. Cochrane Database of Systematic Reviews. 2009, Issue 2. Art. No.: CD005028 |
| 262 | Underdown A; Barlow J; Chung V; Stewart BS. Massage intervention for promoting mental and physical health in infants aged under six months. Cochrane Database of Systematic Reviews. 2009, Issue 1. Art. No.: CD005038 |
| 263 | Jesner OS; Aref AM; Coren E. Risperidone for autism spectrum disorder. Cochrane Database of Systematic Reviews. 2010, Issue 1. Art. No.: CD005040 |
| 264 | Bjornstad GJ; Montgomery P. Family therapy for attention-deficit disorder or attention-deficit/hyperactivity disorder in children and adolescents. Cochrane Database of Systematic Reviews. 2010, Issue 3. Art. No.: CD005042 |
| 265 | Cheuk-Daniel KL; Wong V. Acupuncture for epilepsy. Cochrane Database of Systematic Reviews. 2009, Issue 4. Art. No.: CD005062 |
| 266 | Donnelly D; Everard M; Chang AB. Indoor air modification interventions for prolonged non-specific cough in children. Cochrane Database of Systematic Reviews. 2009, Issue 2. Art. No.: CD005075 |
| 267 | Gibson PG; Powell H; Ducharme F. Long-acting beta2-agonists as an inhaled corticosteroid-sparing agent for chronic asthma in adults and children. Cochrane Database of Systematic Reviews. 2009, Issue 3. Art. No.: CD005076 |
| 268 | Jull AB; Rodgers A; Walker N. Honey as a topical treatment for wounds. Cochrane Database of Systematic Reviews. 2009, Issue 4. Art. No.: CD005083 |
| 269 | Webb AN; Kukuruzovic R; Catto-Smith AG; Sawyer SM. Hypnotherapy for treatment of irritable bowel syndrome. Cochrane Database of Systematic Reviews. 2008, Issue 3. Art. No.: CD005110 |
| 270 | Winzenberg TM; Shaw KA; Fryer J; Jones G. Calcium supplementation for improving bone mineral density in children. Cochrane Database of Systematic Reviews. 2010, Issue 4. Art. No.: CD005119 |
| 271 | Chartapisak W;Opastirakul S;Hodson EM;Willis NS;Craig JC;. Interventions for preventing and treating kidney disease in Henoch-Schönlein Purpura (HSP). Cochrane Database of Systematic Reviews. 2010, Issue 4. Art. No.: CD005128 |
| 272 | Shotton K; Powell C; Voros G; Hatt SR. Interventions for unilateral refractive amblyopia. Cochrane Database of Systematic Reviews. 2009, Issue 3. Art. No.: CD005137 |
| 273 | Zalmanovici A;Yaphe J;. Intranasal steroids for acute sinusitis. Cochrane Database of Systematic Reviews. 2010, Issue 7. Art. No.: CD005149 |
| 274 | Ipser JC;Stein DJ;Hawkridge S;Hoppe L;. Pharmacotherapy for anxiety disorders in children and adolescents. Cochrane Database of Systematic Reviews. 2010, Issue 6. Art. No.: CD005170 |
| 275 | Uman LS; Chambers CT; McGrath PJ; Kisely SR. Psychological interventions for needle-related procedural pain and distress in children and adolescents. Cochrane Database of Systematic Reviews. 2009, Issue 3. Art. No.: CD005179 |
| 276 | McNicol ED; Strassels S; Goudas L; Lau J; Carr DB. NSAIDS or paracetamol, alone or combined with opioids, for cancer pain. Cochrane Database of Systematic Reviews. 2009, Issue 1. Art. No.: CD005180 |
| 277 | Spurling-Geoffrey KP; Fonseka K; Doust J; Del MC. Antibiotics for bronchiolitis in children. Cochrane Database of Systematic Reviews. 2009, Issue 3. Art. No.: CD005189 |
| 278 | Bath-Hextall FJ; Delamere FM; Williams HC. Dietary exclusions for established atopic eczema. Cochrane Database of Systematic Reviews. 2008, Issue 4. Art. No.: CD005203 |
| 279 | Oringanje C;Meremikwu MM;Eko H;Esu E;Meremikwu A;Ehiri JE;. Interventions for preventing unintended pregnancies among adolescents. Cochrane Database of Systematic Reviews. 2010, Issue 1. Art. No.: CD005215 |
| 280 | Gayatri N; Ferrie CD; Cross-Helen HJ. Corticosteroids including ACTH for childhood epilepsy other than epileptic spasms. Cochrane Database of Systematic Reviews. 2009, Issue 1. Art. No.: CD005222 |
| 281 | Glazener-Cathryn MA; Evans-Jonathan HC; Cheuk-Daniel KL. Complementary and miscellaneous interventions for nocturnal enuresis in children. Cochrane Database of Systematic Reviews. 2009, Issue 1. Art. No.: CD005230 |
| 282 | Bhogal SK; Zemek RL; Ducharme F. Written action plans for asthma in children. Cochrane Database of Systematic Reviews. 2009, Issue 1. Art. No.: CD005306 |
| 283 | Ni CM;Greenstone I;Lasserson TJ;Ducharme FM;. Addition of inhaled long-acting beta2-agonists to inhaled steroids as first line therapy for persistent asthma in steroid-naive adults and children. Cochrane Database of Systematic Reviews. 2010, Issue 2. Art. No.: CD005307 |
| 284 | Lasserson TJ; Cates CJ; Lasserson EH; White J. Fluticasone versus 'extrafine' HFA-beclomethasone dipropionate for chronic asthma in adults and children. Cochrane Database of Systematic Reviews. 2010, Issue 2. Art. No.: CD005309 |
| 285 | Chang AB; Halstead-Ria AP; Petsky HL. Methylxanthines for prolonged non-specific cough in children. Cochrane Database of Systematic Reviews. 2009, Issue 3. Art. No.: CD005310 |
| 286 | Vuillermin P; South M; Robertson C. Parent-initiated oral corticosteroid therapy for intermittent wheezing illnesses in children. Cochrane Database of Systematic Reviews. 2009, Issue 2. Art. No.: CD005311 |
| 287 | Ward L; Tricco A; Phuong PN; Cranney A; Barrowman N; Gaboury I; Rauch F; Tugwell P; Moher D. Bisphosphonate therapy for children and adolescents with secondary osteoporosis. Cochrane Database of Systematic Reviews. 2010, Issue 7. Art. No.: CD005324 |
| 288 | Ipser JC; Sander C; Stein DJ. Pharmacotherapy and psychotherapy for body dysmorphic disorder. Cochrane Database of Systematic Reviews. 2009, Issue 1. Art. No.: CD005332 |
| 289 | Tomerak-Ahmed AT; Vyas-Harish HV; Lakhanpaul M; McGlashan J; McKean MC. Inhaled beta2-agonists for non-specific chronic cough in children. Cochrane Database of Systematic Reviews. 2009, Issue 3. Art. No.: CD005373 |
| 290 | Lazzerini M; Ronfani L. Oral zinc for treating diarrhoea in children. Cochrane Database of Systematic Reviews. 2008, Issue 3. Art. No.: CD005436 |
| 291 | Sudarsanam TD; Rupali P; Tharyan P; Abraham OC; Thomas K. Pre-admission antibiotics for suspected cases of meningococcal disease. Cochrane Database of Systematic Reviews. 2008, Issue 4. Art. No.: CD005437 |
| 292 | Ashcroft DM; Chen LC; Garside R; Stein K; Williams HC. Topical pimecrolimus for eczema. Cochrane Database of Systematic Reviews. 2009, Issue 3. Art. No.: CD005500 |
| 293 | Alhashimi D; Al HH; Fedorowicz Z. Antiemetics for reducing vomiting related to acute gastroenteritis in children and adolescents. Cochrane Database of Systematic Reviews. 2009, Issue 2. Art. No.: CD005506 |
| 294 | Carvalho FR; Lentini-Oliveira DA; hado-Marco AC; Saconato H; Prado-Lucila BF; Prado GF. Oral appliances and functional orthopaedic appliances for obstructive sleep apnoea in children. Cochrane Database of Systematic Reviews. 2008, Issue 4. Art. No.: CD005520 |
| 295 | -Bar-On-Edna-;Goldberg E;Fraser A;Vidal L;Hellmann S;Leibovici L;. Combined DTP-HBV-HIB vaccine versus separately administered DTP-HBV and HIB vaccines for primary prevention of diphtheria, tetanus, pertussis, hepatitis B and Haemophilus influenzae B (HIB). Cochrane Database of Systematic Reviews. 2009, Issue 3. Art. No.: CD005530 |
| 296 | Gu R; Shi Y; Wu T; Liu GJ; Zhang M. Chinese medicinal herbs for measles. Cochrane Database of Systematic Reviews. 2010, Issue 7. Art. No.: CD005531 |
| 297 | HemilΣ H; Louhiala P. Vitamin C for preventing and treating pneumonia. Cochrane Database of Systematic Reviews. 2009, Issue 3. Art. No.: CD005532 |
| 298 | Ducharme FM;Ni CM;Greenstone I;Lasserson TJ;. Addition of long-acting beta2-agonists to inhaled steroids versus higher dose inhaled steroids in adults and children with persistent asthma. Cochrane Database of Systematic Reviews. 2010, Issue 4. Art. No.: CD005533 |
| 299 | Ni CM; Greenstone I; Ducharme F; Masse V; Danish A. Long-acting beta2-agonists versus placebo in addition to inhaled corticosteroids in children and adults with chronic asthma. Cochrane Database of Systematic Reviews. 2010, Issue 5. Art. No.: CD005535 |
| 300 | Rodriguez-Martinez CE; Sossa M; Lozano JM. Commercial versus home-made spacers in delivering bronchodilator therapy for acute therapy in children. Cochrane Database of Systematic Reviews. 2009, Issue 1. Art. No.: CD005536 |
| 301 | Omari-Aika AA; Gamble CL; Garner P. Artemether-lumefantrine (six-dose regimen) for treating uncomplicated falciparum malaria. Cochrane Database of Systematic Reviews. 2009, Issue 1. Art. No.: CD005564 |
| 302 | Martφ-Carvajal AJ; Conterno LO. Antibiotics for treating community acquired pneumonia in people with sickle cell disease. Cochrane Database of Systematic Reviews. 2009, Issue 4. Art. No.: CD005598 |
| 303 | Chang AB; Winter D; Acworth JP. Leukotriene receptor antagonist for prolonged non-specific cough in children. Cochrane Database of Systematic Reviews. 2010, Issue 2. Art. No.: CD005602 |
| 304 | Chang AB; Peake J; McElrea MS. Anti-histamines for prolonged non-specific cough in children. Cochrane Database of Systematic Reviews. 2010, Issue 2. Art. No.: CD005604 |
| 305 | Dhiwakar M; Clement WA; Supriya M; McKerrow W. Antibiotics to reduce post-tonsillectomy morbidity. Cochrane Database of Systematic Reviews. 2010, Issue 7. Art. No.: CD005607 |
| 306 | Macfadyen CA; Acuin JM; Gamble CL. Systemic antibiotics versus topical treatments for chronically discharging ears with underlying eardrum perforations. Cochrane Database of Systematic Reviews. 2009, Issue 1. Art. No.: CD005608 |
| 307 | Nwachukwu CE; Okebe JU. Antimotility agents for chronic diarrhoea in people with HIV/AIDS. Cochrane Database of Systematic Reviews. 2008, Issue 4. Art. No.: CD005644 |
| 308 | Lin D; Li WK; Rieder MJ. Cotrimoxazole for prophylaxis or treatment of opportunistic infections of HIV/AIDS in patients with previous history of hypersensitivity to cotrimoxazole. Cochrane Database of Systematic Reviews. 2009, Issue 1. Art. No.: CD005646 |
| 309 | Heirs M; Dean ME. Homeopathy for attention deficit/hyperactivity disorder or hyperkinetic disorder. Cochrane Database of Systematic Reviews. 2009, Issue 1. Art. No.: CD005648 |
| 310 | Foxlee R; Johansson AC; Wejfalk J; Dawkins J; Dooley L; Del MC. Topical analgesia for acute otitis media. Cochrane Database of Systematic Reviews. 2009, Issue 3. Art. No.: CD005657 |
| 311 | Takken T; Van BM; Engelbert-Raoul HH; van-der-Net JJ; Kuis W; Helders-Paul PJM. Exercise therapy in juvenile idiopathic arthritis. Cochrane Database of Systematic Reviews. 2008, Issue 4. Art. No.: CD005954 |
| 312 | Omari-Aika AA; Gamble CL; Garner P. Artemether-lumefantrine (four-dose regimen) for treating uncomplicated falciparum malaria. Cochrane Database of Systematic Reviews. 2010, Issue 1. Art. No.: CD005965 |
| 313 | Graves PM; Gelband H. Vaccines for preventing malaria (SPf66). Cochrane Database of Systematic Reviews. 2009, Issue 2. Art. No.: CD005966 |
| 314 | Rojas M; Granados RC; Charry-Anzola LP. Oxygen therapy for lower respiratory tract infections in children between 3 months and 15 years of age. Cochrane Database of Systematic Reviews. 2009, Issue 3. Art. No.: CD005975 |
| 315 | Haider BA; Saeed MA; Bhutta ZA. Short-course versus long-course antibiotic therapy for non-severe community-acquired pneumonia in children aged 2 months to 59 months. Cochrane Database of Systematic Reviews. 2009, Issue 3. Art. No.: CD005976 |
| 316 | Arnold E; Clark CE; Lasserson TJ; Wu T. Herbal interventions for chronic asthma in adults and children. Cochrane Database of Systematic Reviews. 2008, Issue 4. Art. No.: CD005989 |
| 317 | Moore KN; Fader M; Getliffe K. Long-term bladder management by intermittent catheterisation in adults and children. Cochrane Database of Systematic Reviews. 2009, Issue 1. Art. No.: CD006008 |
| 318 | Effa EE; Bukirwa H. Azithromycin for treating uncomplicated typhoid and paratyphoid fever (enteric fever). Cochrane Database of Systematic Reviews. 2008, Issue 4. Art. No.: CD006083 |
| 319 | Gonzales-Maria LM; Dans LF; Martinez EG. Antiamoebic drugs for treating amoebic colitis. Cochrane Database of Systematic Reviews. 2009, Issue 2. Art. No.: CD006085 |
| 320 | Chang CC; Cheng AC; Chang AB. Over-the-counter (OTC) medications to reduce cough as an adjunct to antibiotics for acute pneumonia in children and adults. Cochrane Database of Systematic Reviews. 2010, Issue 1. Art. No.: CD006088 |
| 321 | Chen H; Zhuo Q; Yuan W; Wang J; Wu T. Vitamin A for preventing acute lower respiratory tract infections in children up to seven years of age. Cochrane Database of Systematic Reviews. 2010, Issue 2. Art. No.: CD006090 |
| 322 | Boyle RJ; Bath-Hextall FJ; Leonardi BJ; Murrell DF; Tang-Mimi LK. Probiotics for treating eczema. Cochrane Database of Systematic Reviews. 2008, Issue 4. Art. No.: CD006135 |
| 323 | Lerch C; Meissner T. Interventions for the prevention of nutritional rickets in term born children. Cochrane Database of Systematic Reviews. 2009, Issue 1. Art. No.: CD006164 |
| 324 | Graves PM; Gelband H. Vaccines for preventing malaria (pre-erythrocytic). Cochrane Database of Systematic Reviews. 2009, Issue 1. Art. No.: CD006198 |
| 325 | Graves PM; Gelband H. Vaccines for preventing malaria (blood-stage). Cochrane Database of Systematic Reviews. 2009, Issue 1. Art. No.: CD006199 |
| 326 | Jefferson T;Del MC;Dooley L;Ferroni E;Al-Ansary LA;Bawazeer GA;van-Driel ML;Nair S;Foxlee R;Rivetti A;. Physical interventions to interrupt or reduce the spread of respiratory viruses. Cochrane Database of Systematic Reviews. 2010, Issue 2. Art. No.: CD006207 |
| 327 | Manning P; Gibson PG; Lasserson TJ. Ciclesonide versus placebo for chronic asthma in adults and children. Cochrane Database of Systematic Reviews. 2008, Issue 4. Art. No.: CD006217 |
| 328 | Mayo WE; Montgomery P; Dennis JA. Personal assistance for children and adolescents (0-18) with physical impairments. Cochrane Database of Systematic Reviews. 2008, Issue 3. Art. No.: CD006277 |
| 329 | Morgan AT; Vogel AP. Intervention for childhood apraxia of speech. Cochrane Database of Systematic Reviews. 2009, Issue 4. Art. No.: CD006278 |
| 330 | Perera R; Haynes J; Glasziou PP; Heneghan CJ. Autoinflation for hearing loss associated with otitis media with effusion. Cochrane Database of Systematic Reviews. 2009, Issue 1. Art. No.: CD006285 |
| 331 | Zhang L; Mendoza-Sassi RA; CΘsar JA; Chadha NK. Intranasal corticosteroids for nasal airway obstruction in children with moderate to severe adenoidal hypertrophy. Cochrane Database of Systematic Reviews. 2009, Issue 1. Art. No.: CD006286 |
| 332 | Rome K;Ashford RL;Evans A;. Non-surgical interventions for paediatric pes planus. Cochrane Database of Systematic Reviews. 2010, Issue 7. Art. No.: CD006311 |
| 333 | Timmer A; Gⁿnther J; Rⁿcker G; Motschall E; Antes G; Kern W. Pelargonium sidoides extract for acute respiratory tract infections. Cochrane Database of Systematic Reviews. 2009, Issue 3. Art. No.: CD006323 |
| 334 | Cates CJ; Cates MJ. Regular treatment with salmeterol for chronic asthma: serious adverse events. Cochrane Database of Systematic Reviews. 2010, Issue 1. Art. No.: CD006363 |
| 335 | Harvey R; Hannan SA; Badia L; Scadding G. Nasal saline irrigations for the symptoms of chronic rhinosinusitis. Cochrane Database of Systematic Reviews. 2009, Issue 1. Art. No.: CD006394 |
| 336 | Vuorio A;Kuoppala J;Kovanen PT;Humphries SE;Strandberg T;Tonstad S;Gylling H;. Statins for children with familial hypercholesterolemia. Cochrane Database of Systematic Reviews. 2010, Issue 7. Art. No.: CD006401 |
| 337 | Shepperd S; Doll H; Gowers S; James A; Fazel M; Fitzpatrick R; Pollock J. Alternatives to inpatient mental health care for children and young people. Cochrane Database of Systematic Reviews. 2009, Issue 2. Art. No.: CD006410 |
| 338 | Gray DM; Zar H; Cotton M. Impact of tuberculosis preventive therapy on tuberculosis and mortality in HIV-infected children. Cochrane Database of Systematic Reviews. 2009, Issue 4. Art. No.: CD006418 |
| 339 | Doan Q;Enarson P;Kissoon N;Klassen TP;Johnson DW;. Rapid viral diagnosis for acute febrile respiratory illness in children in the Emergency Department. Cochrane Database of Systematic Reviews. 2010, Issue 2. Art. No.: CD006452 |
| 340 | Koch MW;Polman-Susanne KL;. Oxcarbazepine versus carbamazepine monotherapy for partial onset seizures. Cochrane Database of Systematic Reviews. 2009, Issue 4. Art. No.: CD006453 |
| 341 | Li Q;Chen X;He L;Zhou D;. Traditional Chinese medicine for epilepsy. Cochrane Database of Systematic Reviews. 2009, Issue 3. Art. No.: CD006454 |
| 342 | Zhang L; Mendoza-Sassi RA; Wainwright C; Klassen TP. Nebulized hypertonic saline solution for acute bronchiolitis in infants. Cochrane Database of Systematic Reviews. 2009, Issue 4. Art. No.: CD006458 |
| 343 | Shotton K; Elliott S. Interventions for strabismic amblyopia. Cochrane Database of Systematic Reviews. 2010, Issue 1. Art. No.: CD006461 |
| 344 | Maas T;Kaper J;Sheikh A;Knottnerus JA;Wesseling G;Dompeling E;Muris-Jean WM;van-Schayck CP;. Mono and multifaceted inhalant and/or food allergen reduction interventions for preventing asthma in children at high risk of developing asthma. Cochrane Database of Systematic Reviews. 2009, Issue 3. Art. No.: CD006480 |
| 345 | Mathew JL; El DR; Mathew PJ; Boxall EH; Brok J. Hepatitis B immunisation in persons not previously exposed to hepatitis B or with unknown exposure status. Cochrane Database of Systematic Reviews. 2009, Issue 1. Art. No.: CD006481 |
| 346 | Goossen GM; Kremer-Leontien CM; van-de-Wetering MD. Influenza vaccination in children being treated with chemotherapy for cancer. Cochrane Database of Systematic Reviews. 2009, Issue 2. Art. No.: CD006484 |
| 347 | Jacquerioz FA;Croft AM;. Drugs for preventing malaria in travellers. Cochrane Database of Systematic Reviews. 2010, Issue 7. Art. No.: CD006491 |
| 348 | Krisanaprakornkit T;Ngamjarus C;Witoonchart C;Piyavhatkul N;. Meditation therapies for attention-deficit/hyperactivity disorder (ADHD). Cochrane Database of Systematic Reviews. 2010, Issue 6. Art. No.: CD006507 |
| 349 | Gregorio G; Gonzales-Maria LM; Dans LF; Martinez EG. Polymer-based oral rehydration solution for treating acute watery diarrhoea. Cochrane Database of Systematic Reviews. 2009, Issue 2. Art. No.: CD006519 |
| 350 | So PS; Jiang Y; Qin Y. Touch therapies for pain relief in adults. Cochrane Database of Systematic Reviews. 2008, Issue 4. Art. No.: CD006535 |
| 351 | Ojukwu JU;Okebe JU;Yahav D;Paul M;. Oral iron supplementation for preventing or treating anaemia among children in malaria-endemic areas. Cochrane Database of Systematic Reviews. 2009, Issue 4. Art. No.: CD006589 |
| 352 | Liu Q; Abba K; Alejandria MM; Balanag VM; Berba RP; Lansang-Mary AD. Reminder systems and late patient tracers in the diagnosis and management of tuberculosis. Cochrane Database of Systematic Reviews. 2008, Issue 4. Art. No.: CD006594 |
| 353 | Bailey EJ; Morris PS; Kruske SG; Chang AB. Clinical pathways for chronic cough in children. Cochrane Database of Systematic Reviews. 2009, Issue 4. Art. No.: CD006595 |
| 354 | Abba K;Gulani A;Sachdev HS;. Zinc supplements for preventing otitis media. Cochrane Database of Systematic Reviews. 2010, Issue 2. Art. No.: CD006639 |
| 355 | Abdelghaffar S; Attia AM. Metformin added to insulin therapy for type 1 diabetes mellitus in adolescents. Cochrane Database of Systematic Reviews. 2009, Issue 1. Art. No.: CD006691 |
| 356 | Nasser M; Fedorowicz Z; Khoshnevisan MH; Shahiri TM. Acyclovir for treating primary herpetic gingivostomatitis. Cochrane Database of Systematic Reviews. 2008, Issue 4. Art. No.: CD006700 |
| 357 | Adetifa I; Okomo U. Iron supplementation for reducing morbidity and mortality in children with HIV. Cochrane Database of Systematic Reviews. 2009, Issue 2. Art. No.: CD006736 |
| 358 | Prince C;David K;John SM;Sankarapandian V;. Antibiotic therapy for Shigella dysentery. Cochrane Database of Systematic Reviews. 2010, Issue 8. Art. No.: CD006784 |
| 359 | Hawke F; Burns J; Radford JA; du T. Custom-made foot orthoses for the treatment of foot pain. Cochrane Database of Systematic Reviews. 2008, Issue 3. Art. No.: CD006801 |
| 360 | Kassel JC;King D;Spurling-Geoffrey KP;. Saline nasal irrigation for acute upper respiratory tract infections. Cochrane Database of Systematic Reviews. 2010, Issue 3. Art. No.: CD006821 |
| 361 | Montgomery P; Mayo WE; Dennis JA. Personal assistance for children and adolescents (0-18) with intellectual impairments. Cochrane Database of Systematic Reviews. 2008, Issue 3. Art. No.: CD006858 |
| 362 | Mayo WE; Montgomery P; Dennis JA. Personal assistance for children and adolescents (0-18) with both physical and intellectual impairments. Cochrane Database of Systematic Reviews. 2008, Issue 3. Art. No.: CD006859 |
| 363 | Cates CJ;Lasserson TJ;Jaeschke R;. Regular treatment with salmeterol and inhaled steroids for chronic asthma: serious adverse events. Cochrane Database of Systematic Reviews. 2009, Issue 3. Art. No.: CD006922 |
| 364 | Cates CJ; Cates MJ; Lasserson TJ. Regular treatment with formoterol for chronic asthma: serious adverse events. Cochrane Database of Systematic Reviews. 2009, Issue 2. Art. No.: CD006923 |
| 365 | Cates CJ; Lasserson TJ; Jaeschke R. Regular treatment with formoterol and inhaled steroids for chronic asthma: serious adverse events. Cochrane Database of Systematic Reviews. 2009, Issue 2. Art. No.: CD006924 |
| 366 | Pennington L;Miller N;Robson S;. Speech therapy for children with dysarthria acquired before three years of age. Cochrane Database of Systematic Reviews. 2010, Issue 1. Art. No.: CD006937 |
| 367 | Nasser M;Fedorowicz Z;Aljufairi H;McKerrow W;. Antihistamines used in addition to topical nasal steroids for intermittent and persistent allergic rhinitis in children. Cochrane Database of Systematic Reviews. 2010, Issue 7. Art. No.: CD006989 |
| 368 | Thomson A; Maltezos S; Paliokosta E; Xenitidis K. Amfetamine for attention deficit hyperactivity disorder in people with intellectual disabilities. Cochrane Database of Systematic Reviews. 2010, Issue 5. Art. No.: CD007009 |
| 369 | Thomson A; Maltezos S; Paliokosta E; Xenitidis K. Risperidone for attention-deficit hyperactivity disorder in people with intellectual disabilities. Cochrane Database of Systematic Reviews. 2010, Issue 5. Art. No.: CD007011 |
| 370 | Manning P; Gibson PG; Lasserson TJ. Ciclesonide versus other inhaled steroids for chronic asthma in children and adults. Cochrane Database of Systematic Reviews. 2009, Issue 3. Art. No.: CD007031 |
| 371 | Cates CJ; Lasserson TJ. Combination formoterol and inhaled steroid versus beta2-agonist as relief medication for chronic asthma in adults and children. Cochrane Database of Systematic Reviews. 2009, Issue 4. Art. No.: CD007085 |
| 372 | Oduwole O;Meremikwu MM;Oyo IA;Udoh EE;. Honey for acute cough in children. Cochrane Database of Systematic Reviews. 2010, Issue 1. Art. No.: CD007094 |
| 373 | Powell G;Saunders M;Marson AG;. Immediate-release versus controlled-release carbamazepine in the treatment of epilepsy. Cochrane Database of Systematic Reviews. 2010, Issue 1. Art. No.: CD007124 |
| 374 | Okwundu C; Okoromah-Christy AN. Antiretroviral pre-exposure prophylaxis (PrEP) for preventing HIV in high-risk individuals. Cochrane Database of Systematic Reviews. 2009, Issue 1. Art. No.: CD007189 |
| 375 | Minozzi S; Amato L; Davoli M. Maintenance treatments for opiate dependent adolescent. Cochrane Database of Systematic Reviews. 2009, Issue 2. Art. No.: CD007210 |
| 376 | Cates CJ; Lasserson TJ. Combination formoterol and budesonide as maintenance and reliever therapy versus inhaled steroid maintenance for chronic asthma in adults and children. Cochrane Database of Systematic Reviews. 2010, Issue 1. Art. No.: CD007313 |
| 377 | Hetrick SE;Purcell R;Garner B;Parslow R;. Combined pharmacotherapy and psychological therapies for post traumatic stress disorder (PTSD). Cochrane Database of Systematic Reviews. 2010, Issue 8. Art. No.: CD007316 |
| 378 | Sinclair D;Zani B;Donegan S;Olliaro P;Garner P;. Artemisinin-based combination therapy for treating uncomplicated malaria. Cochrane Database of Systematic Reviews. 2009, Issue 4. Art. No.: CD007483 |
| 379 | Hillier SL;Louw Q;Morris L;Uwimana J;Statham S;. Massage therapy for people with HIV/AIDS. Cochrane Database of Systematic Reviews. 2010, Issue 1. Art. No.: CD007502 |
| 380 | Mulholland S; Chang AB. Honey and lozenges for children with non-specific cough. Cochrane Database of Systematic Reviews. 2010, Issue 1. Art. No.: CD007523 |
| 381 | Lee RH;Thomas K;Morgan J;Nelson RL;. Lactulose versus Polyethylene Glycol for Chronic Constipation. Cochrane Database of Systematic Reviews. 2010, Issue 7. Art. No.: CD007570 |
| 382 | Wong-May CM;Glenny AM;Tsang-Boyd WK;Lo-Edward CM;Worthington H;Marinho-Valeria CC;. Topical fluoride as a cause of dental fluorosis in children. Cochrane Database of Systematic Reviews. 2010, Issue 6. Art. No.: CD007693 |
| 383 | Cates CJ;Lasserson TJ;. Regular treatment with formoterol and an inhaled corticosteroid versus regular treatment with salmeterol and an inhaled corticosteroid for chronic asthma: serious adverse events. Cochrane Database of Systematic Reviews. 2010, Issue 1. Art. No.: CD007694 |
| 384 | Cates CJ;Lasserson TJ;. Regular treatment with formoterol versus regular treatment with salmeterol for chronic asthma: serious adverse events. Cochrane Database of Systematic Reviews. 2009, Issue 4. Art. No.: CD007695 |
| 385 | Krul M;van-der-Wouden JC;-van-Suijlekom-Smit-Lisette-WA;Koes BW;. Manipulative interventions for reducing pulled elbow in young children. Cochrane Database of Systematic Reviews. 2009, Issue 4. Art. No.: CD007759 |
| 386 | Fedorowicz Z;Al-Muharraqi MA;Nasser M;Al HN;. Oral rinses, mouthwashes and sprays for improving recovery following tonsillectomy. Cochrane Database of Systematic Reviews. 2010, Issue 1. Art. No.: CD007806 |
| 387 | -van-den-Aardweg-Maaike-TA;Schilder-Anne GM;Herkert E;Boonacker-Chantal WB;Rovers MM;. Adenoidectomy for otitis media in children. Cochrane Database of Systematic Reviews. 2010, Issue 5. Art. No.: CD007810 |
| 388 | Walsh T;Worthington H;Glenny AM;Appelbe P;Marinho-Valeria CC;Shi X;. Fluoride toothpastes of different concentrations for preventing dental caries in children and adolescents. Cochrane Database of Systematic Reviews. 2010, Issue 2. Art. No.: CD007868 |
| 389 | Ni CM;Lasserson TJ;Greenstone I;Ducharme FM;. Addition of long-acting beta-agonists to inhaled corticosteroids for chronic asthma in children. Cochrane Database of Systematic Reviews. 2010, Issue 2. Art. No.: CD007949 |
| 390 | Coren E;Hutchfield J;Thomae M;Gustafsson C;. Parent training support for intellectually disabled parents. Cochrane Database of Systematic Reviews. 2010, Issue 6. Art. No.: CD007987 |
| 391 | Zehetner AA;Orr N;Buckmaster A;Williams K;Wheeler DM;. Iron supplementation for breath-holding attacks in children. Cochrane Database of Systematic Reviews. 2010, Issue 7. Art. No.: CD008132 |
| 392 | Odigwe CC;Smedslund G;Ejemot NR;Anyanechi CC;Krawinkel MB;. Supplementary vitamin E, selenium, cysteine and riboflavin for preventing kwashiorkor in preschool children in developing countries. Cochrane Database of Systematic Reviews. 2010, Issue 4. Art. No.: CD008147 |
| 393 | -van-den-Aardweg-Maaike-TA;Schilder-Anne GM;Herkert E;Boonacker-Chantal WB;Rovers MM;. Adenoidectomy for recurrent or chronic nasal symptoms in children. Cochrane Database of Systematic Reviews. 2010, Issue 3. Art. No.: CD008282 |
| 394 | Soares WK;MacLehose H;Ben A;Goldberg E;Pitan F;Cunliffe N;. Vaccines for preventing rotavirus diarrhoea: vaccines in use. Cochrane Database of Systematic Reviews. 2010, Issue 6. Art. No.: CD008521 |
| 395 | Humphreys EH;Smith NA;Azman H;McLeod D;Rutherford GW;. Prevention of diarrhoea in children with HIV infection or exposure to maternal HIV infection. Cochrane Database of Systematic Reviews. 2010, Issue 7. Art. No.: CD008563 |
| 396 | Spaulding A;Rutherford GW;Siegfried N;. Stavudine or zidovudine in three-drug combination therapy for initial treatment of HIV infection in antiretroviral-naïve individuals. Cochrane Database of Systematic Reviews. 2010, Issue 8. Art. No.: CD008651 |
