# Supplementary material for: The Evidence Base for Interventions Delivered to Children in Primary Care: An Overview of Cochrane Systematic Reviews
Source: PLoS One. 2011 Aug 1;6(8):e23051. doi: 10.1371/journal.pone.0023051 (PMC3148227; doi:10.1371/journal.pone.0023051)
Supplement: PRISMA Flow Diagram S1. — (DOC) [file pone.0023051.s005.doc]

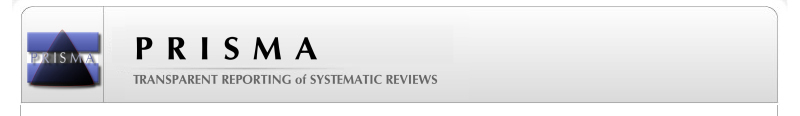
**PRISMA 2009 Flow Diagram**

**Screening**

**Included**

**Eligibility**

**Identification**

Systematic reviews in the CDSR in Issue 8, 2010 identified as child relevant by the Cochrane Child Health Field (n = 1183)

Additional records identified through other sources
(n = 0)

Citations after duplicated removed

(n = 1183)

Systematic reviews screened (n = 1183)

Full-text articles assessed for eligibility
(n = 429)

Full-text articles excluded, with reasons
(n = 33)

Studies included
(n = 396)

Full-text articles excluded (n = 754)
